# Cryoshocked Adipocytes Mediated Dual‐Modal Strategy Combining Photodynamic Therapy and Triptolide Palmitate for Pulmonary Metastatic Melanoma Treatment

**DOI:** 10.1002/advs.202414307

**Published:** 2025-01-13

**Authors:** Aixue Li, Fu Qi, Yuanye Zeng, Rongmei Liu, Huanhuan Cai, Mengyuan He, Dan Li, Yongwei Gu, Jiyong Liu

**Affiliations:** ^1^ Department of Pharmacy Fudan University Shanghai Cancer Center Shanghai 200032 China; ^2^ Department of Oncology Shanghai Medical College Fudan University Shanghai 200032 China; ^3^ College of Pharmacy Shandong University of Traditional Chinese Medicine Jinan Shandong 250355 China; ^4^ Department of Pharmacy Tongji Hospital School of Medicine Tongji University Shanghai 200092 China

**Keywords:** cryoshocked adipocytes, photodynamic therapy, targeted drug delivery, pulmonary metastatic melanoma, triptolide‐palmitate

## Abstract

Pulmonary metastasis represents one of the most prevalent forms of metastasis in advanced melanoma, with mortality rates reaching 70%. Current treatments including chemotherapy, targeted therapy, and immunotherapy frequently exhibit limited efficacy or present high costs. To address these clinical needs, this study presents a biomimetic drug delivery system (Ce6‐pTP‐CsA) utilizing cryoshocked adipocytes (CsA) encapsulating the prodrug triptolide palmitate (pTP) and the photosensitizer Ce6, exploiting the characteristic of tumor cells to recruit and lipolyze adipocytes for energy. CsA substantially enhances the drug‐loading capacity of adipocytes, with its particle size characteristics enabling targeted delivery of pTP to the lungs. The combination of photodynamic therapy (PDT) and pTP activates the caspase cascade, promoting apoptosis in tumor cells. Notably, the cleavage of disulfide bonds in pTP depletes glutathione (GSH), reducing its scavenging effect on reactive oxygen species (ROS) and enhancing the efficacy of PDT. Results demonstrate that Ce6‐pTP‐CsA effectively inhibits the proliferation and invasion of pulmonary metastatic melanoma cells in vitro and induces apoptosis, while significantly suppressing lung metastasis of SCID mice models in vivo. In conclusion, this novel biomimetic drug delivery system based on adipocytes provides a promising strategy for targeted therapy in pulmonary metastatic melanoma.

## Graphical abstract

Lung metastasis is one of the most common forms of metastasis in advanced melanoma, with a mortality rate as high as 70%. This study constructs a biomimetic drug delivery system (Ce6‐pTP‐CsA) based on cryo‐shocked adipocytes loaded with pTP and Ce6, providing a promising strategy for the development of targeted therapeutics for lung metastatic melanoma.

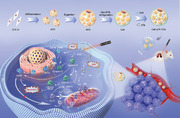

## 1 Introduction

Malignant melanoma is recognized as the most aggressive form of skin cancer, characterized by high invasiveness and significant metastatic potential.^[^ ^1^ ^]^ The 5‐year survival rate for metastatic melanoma remains at only 27%.^[^ ^2^ ^]^ Among the various metastatic sites, pulmonary metastasis is particularly prevalent, with mortality rates reaching 70%.^[^ ^3^ ^]^ While surgical resection is viable for early‐stage melanoma, systemic treatments—including chemotherapy, targeted therapy, and immunotherapy—are the primary interventions for advanced and metastatic cases.^[^ ^2^ ^]^ However, these therapies often exhibit limited efficacy or incur high costs, highlighting the urgent need for targeted formulations effective against metastatic melanoma.

Recent advances in nanoparticle‐based drug carriers with active or passive targeting capabilities have yielded breakthrough achievements in cancer therapy.^[^ ^4^, ^5^, ^6^ ^]^ However, the inherent toxicity of nanoparticles and biological barriers impeding their accumulation at target sites continue to limit drug delivery efficacy.^[^ ^7^ ^]^ These issues pose substantial obstacles to the clinical translation of nanoparticles and have necessitated the exploration of novel drug delivery systems (NDDS). Notably, nano‐bionic drug delivery systems derived from natural cells or their derivatives—including live cells, dead cells, and extracellular vesicles—have emerged as promising candidates in NDDS research.^[^ ^8^, ^9^ ^]^ These systems offer several advantages, including natural targeting ability, biocompatibility, low immunogenicity, intrinsic biodegradability, enhanced biological barrier penetration, and high drug‐loading capacity, enabling reduced immune recognition while improving drug accumulation and efficacy in tumor tissues.^[^ ^10^ ^]^

Extensive research has established the crucial role of adipocytes in tumor cell invasion and metastasis.^[^ ^11^, ^12^ ^]^ Tumor cells actively recruit surrounding adipocytes and induce lipolysis, thereby acquiring energy from long‐chain fatty acids through membrane lipid transport proteins, which in turn supports tumor cell metabolism.^[^ ^13^ ^]^ Furthermore, tumor cells require substantial lipid materials, such as phospholipids for cell membrane biogenesis, to maintain rapid proliferation.^[^ ^14^ ^]^ Consequently, the utilization of adipocytes as drug carriers exploiting lipid metabolism pathways has attracted considerable attention.^[^ ^12^, ^15^ ^]^ However, tumor cells can reprogram adipocytes into tumor‐associated adipocytes facilitating metastasis, and living cells exhibit limited drug‐loading capacity. To overcome these limitations, the cell cryoshock technique which employs rapid freeze‐thaw cycles with liquid nitrogen, has been developed to produce cells that maintain protein‐mediated biological functions while losing cellular activity.^[^ ^16^, ^17^ ^]^ Cryoshocked adipocytes (CsA) exhibit several advantages as drug delivery carriers. First, the active recruitment of adipocytes by tumor cells, coupled with the optimal particle size of adipocytes, enhances targeted pulmonary drug delivery. Second, tumor‐recruited CsA can disrupt interactions between tumor cells and normal adipocytes, thereby impeding tumor progression.^[^ ^18^ ^]^ Additionally, CsA mitigate potential risks associated with live cell therapies while significantly enhancing drug‐loading capacity.

Our preliminary research has demonstrated that triptolide (TP) exhibits potent anti‐melanoma effects but is limited by a short half‐life and significant hepatotoxicity and nephrotoxicity.^[^ ^19^, ^20^ ^]^ By modifying TP with palmitic acid (PA) via a disulfide bond, we have synthesized the prodrug triptolide‐palmitate (pTP), which can exploit the fatty acid uptake mechanisms of tumor cells, promoting active drug intake and extending half‐life, thus enhancing efficacy while reducing toxicity (Figure S1, Supporting Information). Malignant tumors achieve rapid proliferation and invasion through multiple pathways, including immune evasion and the development of drug resistance. While monotherapy is often insufficient for effective suppression, combination therapy has demonstrated significant efficacy in the clinical treatment of metastatic or advanced tumors.^[^ ^21^, ^22^ ^]^ Photodynamic therapy (PDT), which utilizes singlet oxygen produced by light‐activated photosensitizers to induce apoptosis, frequently complements surgery, radiotherapy, and chemotherapy.^[^ ^23^ ^]^

In summary, based on principles of compatibility and hyperosmolarity, we have developed a biomimetic drug delivery system (Ce6‐pTP‐CsA) by encapsulating pTP and the photosensitizer Ce6 within CsA for targeted therapy against pulmonary metastatic melanoma. Adipocytes recruited by tumor tissue undergo lipolysis, releasing both pTP and Ce6. Within the tumor microenvironment, high glutathione (GSH) concentrations trigger disulfide bond cleavage, releasing TP and activating mitochondrial apoptosis pathways. Simultaneously, GSH depletion enhances Ce6's photodynamic effects, accelerating reactive oxygen species (ROS) production and triggering endoplasmic reticulum stress pathways. Furthermore, ROS‐induced mitochondrial damage promotes the TP‐induced caspase cascade response, resulting in efficient and sustained tumor cell apoptosis. Through comprehensive in vivo and in vitro experiments, we have systematically validated the mechanism of action of Ce6‐pTP‐CsA on A375‐M1 cells, while demonstrating its synergistic antitumor effects and underlying mechanisms. This cryoshocked adipocyte‐mediated drug delivery approach represents a promising combination therapy strategy with significant potential for clinical application in improving the treatment outcome for pulmonary metastatic melanoma.

## 2 Results and Discussion

### 2.1 Preparation and Characterization of Ce6‐pTP‐CsA

**Figure 1:**
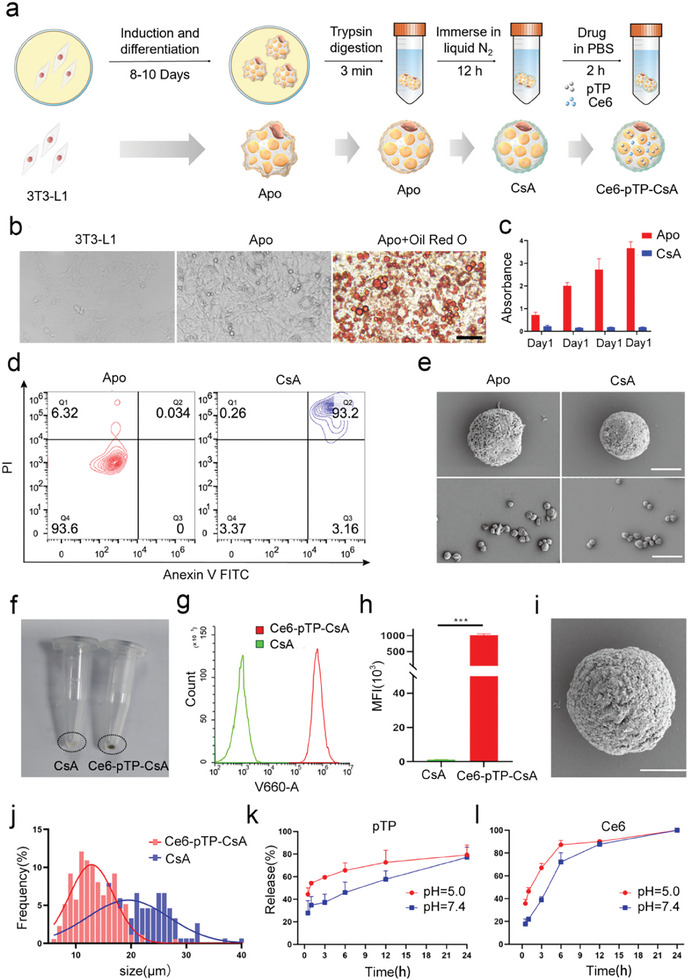
Preparation and characterization of Ce6‐pTP‐CsA. a) Schematic diagram of the preparation process of Ce6‐pTP‐CsA. b) Inverted microscope images of 3T3‐L1 cells, mature adipocytes (Apo) after induction, and Apo stained with Oil Red O. Scale bar: 50 µm. c) CCK‐8 assay analysis of activity for Apo and CsA (n = 3). d) Apoptosis analysis of CsA using Annexin V‐FITC/PI staining (n = 3). e) Representative SEM images of Apo and CsA. Scale bar: 5 µm. f) Visual comparison of CsA and Ce6‐pTP‐CsA in a 1.5 mL EP tube. g) CLSM of CsA and h) histogram comparing the MFI (n = 3). i) Representative SEM image of Ce6‐pTP‐CsA. Scale bar: 5 µm. j) Particle size distribution of CsA and Ce6‐pTP‐CsA. Release curves of k) pTP and l) Ce6 from Ce6‐pTP‐CsA in media at pH 5 and pH 7.4 (n = 3). (***p < 0.001).

Adipocytes, as mature differentiated cells, are generated by the activation of preadipocytes.^[^ ^24^ ^]^ Specifically, the activation of 3T3‐L1 cells via insulin stimulates the insulin receptor substrate 1 (IRS1), PI3K, and AKT1 or AKT2 kinases, which further activate downstream signaling proteins, including CREB, mTOR, and the FOXO family, ultimately leading to the formation of mature adipocytes with distinct lipid droplets (**Figure** **1**a). The successful differentiation of adipocytes was confirmed through Oil Red O staining, which revealed numerous reddish‐brown lipid droplets in each cell (Figure 1b). Subsequently, CsA was prepared using a liquid nitrogen immersion technique. This method ensured the inactivation of cells while preserving their proteins and morphology through the selection of an appropriate cryoprotectant. Compared to the traditional gradual cooling method (storing cells in a gradient freezing box at −80 °C for 6 h before transferring them to liquid nitrogen for another 6 h), directly immersing live cells mixed with 10–20% DMSO into liquid nitrogen results in a significant decrease in cell viability (Figure S2, Supporting Information). Furthermore, when the DMSO concentration in the cryoprotectant was reduced to less than 2%, the absorbance reached a minimum and stabilized (Figure S3, Supporting Information). To ensure minimal cellular activity, a cryoprotectant containing 1% DMSO was selected for CsA preparation, which was subsequently used in further experiments.

To confirm whether CsA cells had indeed become inactive and entered a state of cryogenic shock, apoptosis and cytotoxicity assays were performed. Flow cytometry (FCM) analysis of apoptosis demonstrated that CsA cells subjected to liquid nitrogen immersion had almost completely lost their viability, with only 3.4% ± 1.2% of cells remaining viable (Figure 1d). The CCK‐8 assay results further supported this finding, as CsA exhibited consistently low absorbance values over four consecutive days, indicating that the cells had undergone shock‐induced death during the liquid nitrogen immersion process (Figure 1c).^[^ ^17^, ^25^ ^]^ Electron microscopy demonstrated that CsA retained its basic cellular structure, with an average particle size of 18.7 ± 8.1 µm (Figure 1e; Figure S4, Supporting Information). These results collectively confirm the successful preparation of CsA.

The optimal loading conditions were determined using encapsulation efficiency and drug loading capacity as evaluation indicators. The optimal concentrations were established at 500 µg·mL^−1^ for pTP and 50 µg·mL^−1^ for Ce6, with an optimal incubation time of 2 h for both drugs (Figures S5 and S6, Supporting Information). In the co‐encapsulation system, pTP achieved a drug loading capacity of 22.97 ± 0.96 µg/10^6^ cells and an encapsulation efficiency of 4.59 ± 0.19%, while Ce6 exhibited values of 2.88 ± 0.19 µg/10^6^ cells and 5.75% ± 0.39%, respectively. Subsequently, the drug delivery system Ce6‐pTP‐CsA was successfully constructed by incubating CsA with PBS containing both pTP and Ce6.

The appearance of Ce6‐pTP‐CsA was light gray (Figure 1f), attributable to the black color of Ce6, which altered the appearance of CsA upon drug loading. Figure 1g,h further confirm the successful encapsulation of Ce6 within CsA. Electron microscopy revealed that Ce6‐pTP‐CsA was spherical in shape, with a sparsely porous surface and a granular texture (Figure 1i). Particle size analysis indicated that the average particle size of CsA was 19.5 ± 7.4 µm, while that of Ce6‐pTP‐CsA was 12.9 ± 4.1 µm (Figure 1j). Similar results were observed under a bright‐field inverted microscope (Figure S7, Supporting Information). Additionally, forward scatter (FSC) and side scatter (SSC) data from FCM were used to assess cell size and the number of intracellular particles.^[^ ^17^ ^]^ The results showed that the particle size of Ce6‐pTP‐CsA was reduced compared to CsA, with a decrease in intracellular particles, likely due to the leakage of some cellular contents during incubation (Figure S8, Supporting Information). Finally, total protein identification demonstrated that the protein bands in the Apo (adipocytes), CsA, and Ce6‐pTP‐CsA groups were largely consistent, indicating no significant loss of proteins (Figure S9, Supporting Information).^[^ ^16^ ^]^ This finding suggests that during the preparation of CsA and Ce6‐pTP‐CsA, most of the proteins in the Apo were retained, ensuring that the molecular mechanisms underlying the interaction between A375‐M1 cells and CsA were preserved.

To assess the release rate and extent of Ce6‐pTP‐CsA at the tumor site, in vitro release experiments were conducted. pTP exhibited a burst release at pH 5.0, with 44.43% ± 5.67% released within 0.5 h (Figure 1k), likely due to the acidic environment deforming the phospholipid bilayer and accelerating drug release. This observation supports the rationale for drug release in the acidic tumor microenvironment.^[^ ^26^ ^]^ Similarly, Ce6 exhibited sustained release at pH 7.4 but underwent a burst release at pH 5.0. After 12 h, the cumulative release in both groups reached 87.65 ± 2.00 µg·mL^−1^ and 90.07 ± 0.20 µg·mL^−1^, respectively, indicating that Ce6 was nearly completely released within 12 h. Therefore, light irradiation should be applied promptly after administration to maximize the efficacy of PDT (Figure 1l).

### 2.2 Cellular Uptake of Ce6‐pTP‐CsA

**Figure 2:**
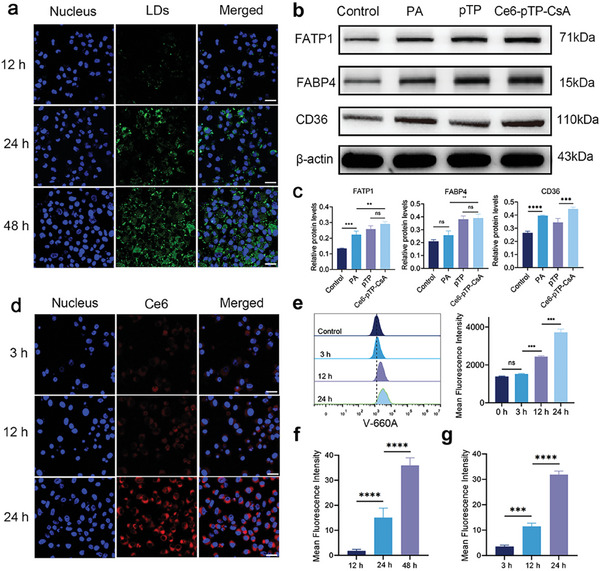
Cellular uptake of Ce6‐pTP‐CsA. a) Representative CLSM images and f) analysis of MFI showing the cellular uptake of BODIPY‐stained Ce6‐pTP‐CsA co‐incubated with A375‐M1 cells for 12 h, 24 h, and 48 h (n = 3). Scale bar: 50 µm. b,c) Western blot analysis of FATP1, FABP4, and CD36 expression in A375‐M1 cells treated with PBS, PA, pTP, and Ce6‐pTP‐CsA (n = 3). d) CLSM and g) MFI analysis of Ce6 uptake in A375‐M1 cells for 3, 12, and 24 h (n = 3). Scale bar: 20 µm (n = 3). e) FCM analysis of Ce6 uptake in A375‐M1 cells for different durations (n = 3). (ns, not significant; **p < 0.01; ***p < 0.001; ****p < 0.0001).

The recruitment and lipolysis of Ce6‐pTP‐CsA by A375‐M1 cells are crucial for CsA to serve as a carrier for delivering drugs to tumor tissues. Following the co‐incubation of BODIPY 505/515‐stained CsA with A375‐M1 cells, the cells internalized CsA and underwent lipolysis. The mean fluorescence intensity (MFI) in A375‐M1 cells increased over time, and after 12 h, the fluorescence intensity of lipid droplets within tumor cells exceeded that of the cell membrane. This could be attributed to the internalization of CsA into lipid droplets by A375‐M1 cells (**Figure** **2**a,f).^[^ ^27^ ^]^ To further investigate the mechanism of CsA lipolysis in A375‐M1 cells, PA was used as a positive control, confirming that PA upregulated proteins are involved in fatty acid transport. In the Ce6‐pTP‐CsA group, compared to the control group, fatty acid transport protein 1 (FATP1), fatty acid‐binding protein 4 (FABP4), and CD36 were upregulated by 2.17 ± 0.10, 2.12 ± 0.14, and 1.69 ± 0.05‐fold, respectively. The FATP family is involved in the transport, metabolism, and accumulation of fatty acids within cells.^[^ ^28^ ^]^ The FABP family plays a key role in the uptake, transport, and metabolic regulation of long‐chain fatty acids, facilitating the solubilization, transport, and metabolism of fatty acids within cells. Moreover, the transporter CD36 mediates the transport of PA.^[^ ^29^ ^]^ These results demonstrate that the uptake of pTP and the breakdown of Ce6‐pTP‐CsA in A375‐M1 cells involve the participation of proteins such as FATP1, FABP4, and CD36 (Figure 2b,c).

Leveraging the fluorescent properties of Ce6, the internalization of Ce6 in A375‐M1 cells was tracked via confocal laser scanning microscopy (CLSM).^[^ ^30^ ^]^ The results indicated that A375‐M1 cells internalized Ce6 from Ce6‐pTP‐CsA, and the uptake increased over time (Figure 2d,g). FCM results corroborated these findings. The uptake of Ce6 from Ce6‐pTP‐CsA was 1.10 ± 0.02, 1.76 ± 0.03, and 2.68 ± 0.13‐fold that of the control group at 3, 12, and 24 h, respectively (Figure 2e). Furthermore, by adding Ce6‐pTP‐CsA to either the upper or lower chambers of the Transwell plate, we further confirmed that Ce6 was mostly taken up when Ce6‐pTP‐CsA was in direct contact with A375M1 cells. This indicates that the drug is released through the lipolysis of Ce6‐pTP‐CsA rather than via passive diffusion (Figure S10, Supporting Information).

To verify the specificity and safety of Ce6‐pTP‐CsA, we co‐incubated Ce6‐pTP‐CsA with human bronchial epithelial cells (BEAS‐2B) and macrophages (RAW264.7) to observe the uptake behavior. The results showed that lung cells exhibited almost no uptake of Ce6‐pTP‐CsA, while macrophages displayed only a small amount of uptake, which was significantly lower than that of A375M1 cells (Figure S11, Supporting Information). Therefore, Ce6‐pTP‐CsA is minimally affected by off‐target effects and demonstrates both specificity and safety in tumor therapy.

### 2.3 In Vitro Antitumor Effect of Ce6‐pTP‐CsA

First, the transformation of pTP in the tumor microenvironment was simulated to determine the rate at which pTP converts to TP. In the simulated tumor microenvironment group (pH = 5.0 + 10 mM GSH), pTP underwent substantial conversion within a short time frame, with a conversion rate of ≈276.7 nmol·h^−1^ during the first 2 h. In the simulated physiological environment group (1 µM, pH = 7.4), pTP conversion occurred slowly, indicating good safety. The acidic control group (1 µM, pH = 5.0) also demonstrated that pTP conversion is not hindered by the low pH of the tumor microenvironment (Figure S12, Supporting Information). Taken together, pTP can rapidly convert to the active compound in the acidic tumor microenvironment with high GSH concentrations, exerting antitumor effects.

**Figure 3:**
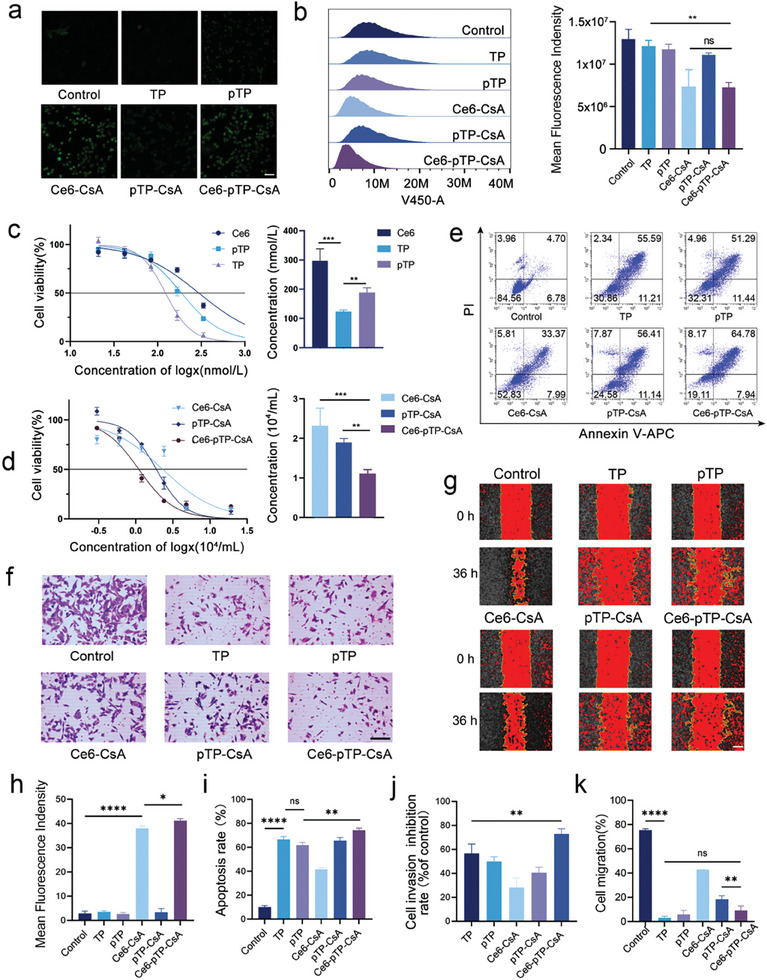
In vitro antitumor effect. a) CLSM and h) MFI of ROS in A375‐M1 cells reacting with H2DCFDA (n = 3). Scale bar: 100 µm. b) Flow cytometric analysis of DPBF reacting with ROS in A375‐M1 cells (n = 3). c,d) Relative cell viability and IC50 values of A375‐M1 cells treated with Ce6, TP, pTP, Ce6‐CsA, pTP‐CsA, and Ce6‐pTP‐CsA (n = 3). e,i) Apoptosis rates of A375‐M1 cells treated with different formulations (n = 3). f,j) Transwell images of A375‐M1 cell invasion after different treatments (n = 3). g,k) Representative images of A375‐M1 cell migration after various treatments (n = 3). (ns, not significant; *p < 0.1; **p < 0.01; ***p < 0.001; ****p < 0.0001).

Subsequently, we assessed the PDT efficacy of Ce6‐pTP‐CsA based on its ROS generation capacity. CLSM results indicated that the MFI in the pTP group was significantly higher than that in the TP group. This is likely due to the conversion of pTP to TP, which consumes GSH and then reduces GSH‐mediated ROS clearance, thereby enhancing the effectiveness of PDT. Both the Ce6‐CsA and Ce6‐pTP‐CsA groups exhibited strong fluorescence, indicating that the PDT effect of Ce6 can induce significant ROS production in A375‐M1 cells, leading to cell necrosis and apoptosis (**Figure** **3**a,h). Notably, the MFI of the Ce6‐pTP‐CsA group was significantly greater than that of the Ce6‐CsA group, as the conversion of pTP to TP reduced GSH's scavenging effect on ROS, while TP also promoted ROS production in A375‐M1 cells, further enhancing the efficacy of PDT. Subsequent quantitative analysis of ROS production was conducted using the fluorescent probe DPBF through FCM. When DPBF encounters ROS, the molecular structure of its barbituric acid moiety reacts with ROS, resulting in fluorescence quenching and a subsequent decrease in fluorescence intensity.^[^ ^31^ ^]^ As shown in Figure 3b, the fluorescence signal intensity at 410 nm significantly decreased in the Ce6‐CsA and Ce6‐pTP‐CsA groups, measuring 0.65 ± 0.09 and 0.51 ± 0.05 times that of the control group, respectively. The signal intensity of the Ce6‐pTP‐CsA group was only 0.79 ± 0.03 times that of the Ce6‐CsA group, indicating that Ce6‐pTP‐CsA can effectively activate endoplasmic reticulum stress signaling pathways to mediate A375‐M1 cell death.

Through in vitro experiments assessing cytotoxicity, apoptosis, invasion, and wound healing, we examined the synergistic killing effects of Ce6‐pTP‐CsA on A375‐M1 cells. The cytotoxicity results revealed IC_50_ values for Ce6, TP, and pTP of 296.8 ± 41.5, 123.3 ± 6.0, and 187.9 ± 16.9 nmol·L^−1^, respectively (Figure 3c). The IC_50_ value of the TP group was significantly lower than that of the pTP group, possibly due to the incomplete conversion of pTP to TP in the in vitro environment, which does not accurately simulate the tumor microenvironment in vivo.^[^ ^32^ ^]^ The cytotoxicity of the Ce6‐pTP‐CsA group was significantly higher than the single‐drug‐loaded formulation group, demonstrating the synergistic antitumor proliferative effects of TP combined with PDT (Figure 3d). Further analysis of apoptosis indicated that the highest apoptosis rate was achieved with Ce6‐pTP‐CsA treatment in A375‐M1 cells, reaching 74.36% ± 1.61% (Figure 3e,i). This may be related to both the synergistic activation of caspase‐independent apoptosis signaling pathways by PDT and TP, as well as the ROS‐mediated induction of apoptosis, necrosis, and autophagy in tumor cells.^[^ ^33^, ^34^ ^]^ The results of the transwell assays further indicated that Ce6‐pTP‐CsA had the most significant impact on cell invasion, achieving an invasion inhibition rate of 72% ± 4.8% (Figure 3f,j). The wound healing assays yielded similar results. While there was no significant difference in the inhibitory effects of Ce6‐pTP‐CsA and free TP on tumor cell migration, it is important to note that TP cannot persist in the in vivo environment for extended periods, suggesting that Ce6‐pTP‐CsA would exhibit a more pronounced migration inhibitory effect in vivo (Figure 3g,k).

### 2.4 Synergistic Mechanism

**Figure 4:**
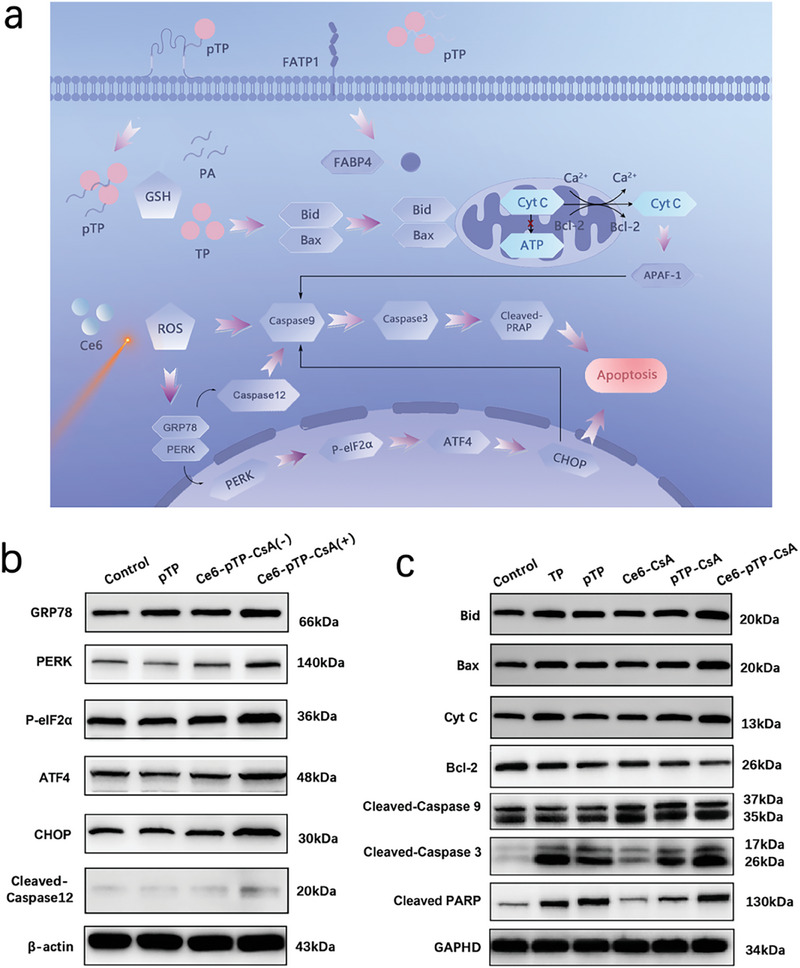
Synergistic mechanism. a) Schematic illustration of the synergistic effect of Ce6‐pTP‐CsA in inducing apoptosis in A375 cells. b) Western blot analysis of CGRP78, PERK, P‐eIF2*α*, ATF4, CHOP, and Cleaved‐Caspase 12 expression in A375‐M1 cells after treatment with PBS, pTP and Ce6‐pTP‐CsA (±hv) (n = 3). c) Western blot analysis of Bax, Bid, Cytochrome C, Bcl‐2, Cleaved‐Caspase 9, Cleaved‐Caspase 3, and Cleaved PARP expression in A375‐M1 cells after treatment with PBS, TP, pTP, Ce6‐CsA, pTP‐CsA, and Ce6‐pTP‐CsA (n = 3).

Ce6‐pTP‐CsA exhibited excellent synergistic antitumor activity, prompting further investigation into its potential mechanism (**Figure** **4**a). Studies have shown that the excessive ROS generated by PDT can induce endoplasmic reticulum stress which is considered a major contributor to apoptosis.^[^ ^35^ ^]^ Western blot results demonstrated that both pTP and non‐irradiated Ce6‐pTP‐CsA did not activate the endoplasmic reticulum stress pathway. However, in laser‐irradiated Ce6‐pTP‐CsA, the expression levels of PERK, P‐eIF2*α*, ATF4, and CHOP were upregulated by 1.86 ± 0.13, 1.39 ± 0.07, 1.52 ± 0.09, and 1.87 ± 0.08‐fold, respectively, compared to the control group (Figure 4b; Figure S13, Supporting Information). Ce6 is activated by light of a specific wavelength, leading to the generation of ROS, which triggers the release and activation of the endoplasmic reticulum stress protein PERK from GRP78. Meanwhile, Caspase 12 is activated to Cleaved‐Caspase 12, triggering a cascade reaction that ultimately leads to cell apoptosis. PERK catalyzes the phosphorylation of eIF2*α* (P‐eIF2*α*). Simultaneously, P‐eIF2*α* regulates translation, influencing ATF4, which in turn upregulates CHOP, ultimately leading to apoptosis.^[^ ^36^ ^]^

pTP primarily promotes apoptosis through the mitochondria‐involved endogenous apoptotic pathway. As shown in Figure 4c and Figure S14 (Supporting Information), pTP stimulates Bid to directly bind with Bax, promoting the translocation of Bax from the cytoplasm to the mitochondrial outer membrane and its activation. The activated Bax forms channels, increasing the permeability of the mitochondrial membrane, thereby facilitating the release of apoptotic factors such as Cytochrome C (Cyt C) from the mitochondria into the cytoplasm. The loss of Cyt C disrupts the respiratory chain, impeding ATP production, and initiating mitochondrial apoptosis.^[^ ^37^ ^]^ Meanwhile, Cyt C binds to Apaf‐1 in the cytoplasm to form an apoptosome, which recruits Caspase 9 zymogen, promoting its dimerization and generating proteolytic signals. This leads to self‐cleavage, activating Cleaved‐Caspase 9, which in turn triggers a caspase cascade, resulting in the activation of Caspase 3 into Cleaved‐Caspase 3, which exerts its proteolytic function and induces apoptosis. Finally, cleaved PARP is activated directly or indirectly by Caspase 3, executing apoptosis.^[^ ^38^, ^39^ ^]^

Notably, the excessive ROS produced by PDT can disrupt the mitochondrial membrane, facilitating the release of Cyt C and further enhancing the caspase cascade process. Additionally, studies have shown that the overexpression of CHOP exerts a negative regulatory effect on Bcl‐2, leading to the translocation of Bax from the cytoplasm to the mitochondria, initiating the mitochondrial apoptotic pathway and enhancing apoptosis.^[^ ^40^, ^41^ ^]^ These findings provide support for the synergistic anti‐tumor effects of PDT combined with pTP.

### 2.5 Construction of the Mouse Melanoma Pulmonary Metastasis Model

**Figure 5:**
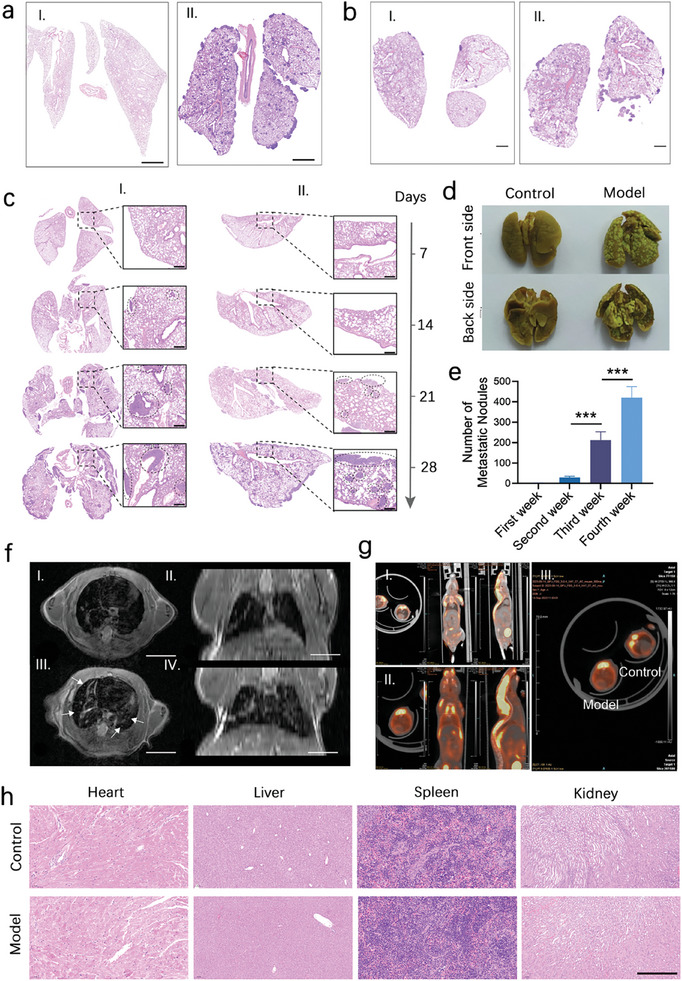
Establishment of the SCID mouse melanoma lung metastasis model. a) H&E slices of the lungs of BALB/C nude mice and SCID mice after tail vein injection of A375‐M1 cells. Scale bar: 2 mm. b) H&E slices of the lungs of SCID mice after injection of A375‐M1 cells at concentrations of 1 × 10^7^ cells·mL^−1^ and 2 × 10^7^ cells·mL^−1^. Scale bar: 2 mm. c) Weekly H&E staining results of the lungs of SCID mouse model. Scale bar: 200 µm. d) Bouin's fixation staining images of the lungs of SCID mice and e) statistical analysis of surface nodules in the lungs (n = 5). f) MRI images of SCID mouse model (I and II represent cross‐sectional and coronal MRI images of control group SCID mice; III and IV represent cross‐sectional and coronal MRI images of model group SCID mice). Scale bar: 1 cm. g) PET‐CT imaging of SCID mice (I: PET‐CT images of the lungs in the blank control group; II: PET‐CT images of the lungs in the model group). h) H&E slices of major organs of SCID mice after 4 weeks of modeling. Scale bar: 200 µm. (***p < 0.001).

To investigate the biodistribution and antitumor effects of Ce6‐pTP‐CsA in vivo, we first developed the method for establishing a melanoma pulmonary metastasis model in mice. Through optimization of the mouse strain and cell inoculation parameters, we established a melanoma pulmonary metastasis model using SCID mice via tail vein injection of 100 µL of A375‐M1 cells at a concentration of 2 × 10⁷ cells·mL^−1^ (**Figure** **5**a,b). Following model establishment, weekly histological analyses using lung H&E staining and nodule quantification were conducted. The results revealed the progressive development of metastatic foci with a characteristic satellite distribution starting from the second week, predominantly occurring on the lung surface and near perivasculature regions.^[^ ^2^, ^42^ ^]^ By the fourth week, the nodule count reached 420 ± 55 (Figure 5c‐e).

Magnetic resonance imaging (MRI) revealed multiple abnormal signals in the lungs of model mice at 4 weeks post‐modeling. The metastatic foci, indicated by arrows, exhibited significantly abnormal signal intensities with enhanced brightness and well‐defined lesion boundaries (Figure 5f).^[^ ^43^, ^44^ ^]^ Additionally, positron emission tomography/computed tomography (PET/CT) demonstrated a diffuse distribution of the contrast agent in the lungs, with higher signal intensity in the peripheral regions compared to the central lung parenchyma (Figure 5g). Notably, throughout the 4‐week modeling period, no significant differences in body weight were observed between control and model mice (Figure S15, Supporting Information). And four‐week histological examination and anatomical inspections showed no evidence of tumor metastases in organs other than the lungs (Figure 5h). These results further confirm the successful establishment of the melanoma pulmonary metastasis model in mice.

### 2.6 Biodistribution

**Figure 6:**
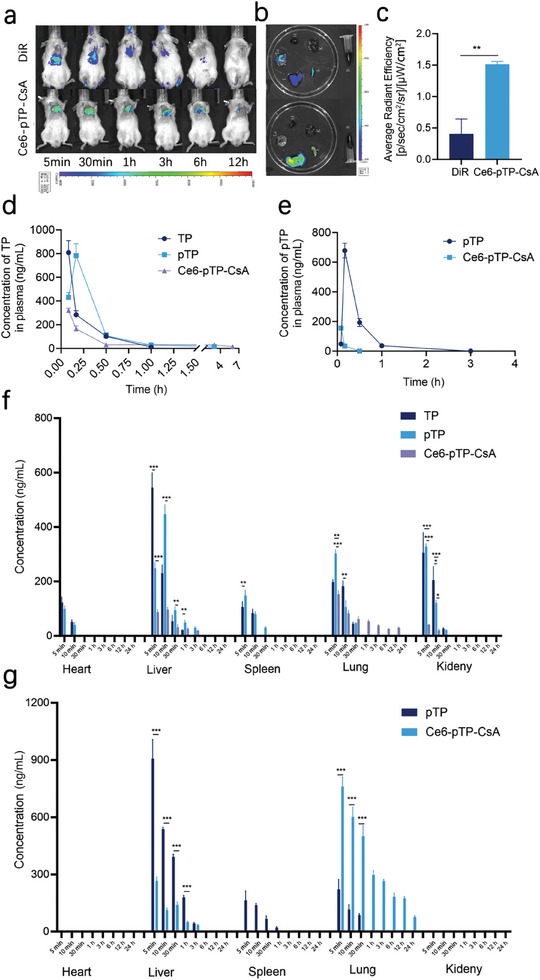
Biodistribution. a) Real‐time fluorescence images and b) ex vivo organ imaging after intravenous injection of DiR and DiR‐labeled Ce6‐pTP‐CsA in SCID mouse model. c) Radiant efficiency in the lungs after injection of DiR and DiR‐labeled Ce6‐pTP‐CsA for 12 h (n = 3). d) Plasma concentration‐time curves of TP and e) pTP in SCID mouse model (n = 3). Changes in f) TP and g) pTP levels in major organs after injection of TP, pTP, and Ce6‐pTP‐CsA (TP dose of 0.6 mg·kg^−1^) (n = 3). (ns, not significant; *p < 0.1; **p < 0.01; ***p < 0.001).

Enhancement of drug accumulation in vivo represents a critical factor determining therapeutic efficacy. In vivo imaging results showed that free DIR diffused freely throughout the body with the bloodstream, resulting in almost complete fluorescence disappearance after 3 h. In contrast, the DiR‐labeled Ce6‐pTP‐CsA exhibited selective localization in the lungs, demonstrating strong fluorescence intensity and prolonged duration, with substantial signal persisting at 12 h (**Figure** **6**a). Ex vivo organ imaging further revealed that Ce6‐pTP‐CsA displayed strong fluorescence in the lungs, with an intensity 3.73 ± 0.11 times that of the DIR group, confirming the lung‐targeting capability of Ce6‐pTP‐CsA (Figure 6b,c). This enhanced lung targeting can be attributed to the active recruitment of adipocytes by tumor cells and the specific size characteristics of these adipocytes.

Subsequently, the biodistribution of Ce6‐pTP‐CsA was further evaluated using HPLC‐MS/MS. As shown in Figure 6f, Ce6‐pTP‐CsA facilitated effective TP accumulation in the lungs through passive targeting. Moreover, TP was still detectable in the lungs at 12 h, representing an ≈20‐fold increase in retention compared to free TP, thus effectively addressing the short half‐life limitation of TP. This enhanced retention may be attributed to the protective effect of CsA on pTP, preventing its enzymatic metabolism such as CYP3A4, and enabling sustained release for continuous antitumor effects. Additionally, Ce6‐pTP‐CsA modified the systemic distribution of TP, showing minimal cardiac and splenic accumulation, and significantly reduced hepatic and renal concentrations, suggesting reduced hepatotoxicity and nephrotoxicity. The pulmonary pharmacokinetic parameters of Ce6‐pTP‐CsA demonstrated that its T_1/2_ value was ≈18‐fold that of the free drug group, with the AUC(0‐t) value exhibiting a fourfold enhancement, indicating enhanced accumulation of TP in the target organ, which theoretically could lead to improved tumor inhibition effects (Table S1, Supporting Information).

To elucidate the transformation and distribution of pTP within tissues, further analyses were conducted. As shown in Figure 6g, pTP exhibited minimal cardiac and renal accumulation. Combined with the tissue distribution results of TP, these findings suggest that abundant cardiac blood flow promotes oxidase‐mediated metabolism of pTP's disulfide bonds, leading to rapid TP accumulation. Additionally, pTP may undergo complete metabolism to TP before reaching the kidneys. While free TP accumulation in the liver and spleen could potentially induce organ damage, CsA modulation of drug distribution promotes accumulation around the target organ. These findings underscore the importance of CsA in pTP delivery for reducing bloodstream metabolism and enhancing tumor site accumulation.^[^ ^45^, ^46^ ^]^

Moreover, pTP extended the drug's bloodstream metabolic duration, approximately doubling the circulation T_1/2_ of TP. Ce6‐pTP‐CsA enables controlled TP release, maintaining stable bloodstream concentrations, with a ≈50% increase in AUC_(0‐t)_ compared to free TP, effectively delaying metabolism (Figure 6d,e; Table S2, Supporting Information).^[^ ^47^ ^]^ In conclusion, the approach of using CsA to deliver the lipophilic prodrug pTP successfully achieves the intended lung targeting and sustained release objectives.

### 2.7 Therapeutic Effect In Vivo

**Figure 7:**
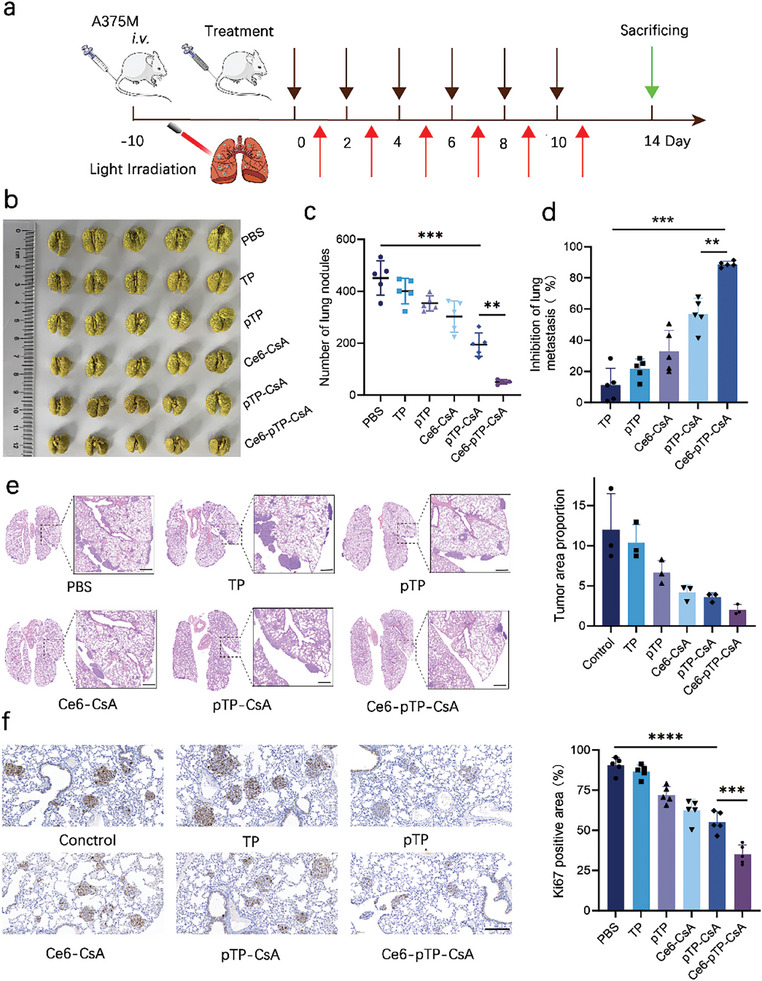
Therapeutic efficacy in vivo. a) Schematic diagram of treatment strategy. Following 12 h post‐administration, phototherapy was applied to the lungs of mice (power: 50 mW, wavelength: 660 nm, irradiation duration: 5 min). b) Representative Bouin's staining images of pulmonary tissue. c) Quantitative analysis of pulmonary surface metastatic lesions (n = 5). d) Statistical analysis of metastatic inhibition rates (n = 5). e) H&E staining of lung slices and proportion of metastatic lesion area. Scale bar: 500 µm (n = 3). f) Ki67 staining and quantification of positive rates in lung tissue. Scale bar = 200 µm (n = 5). (ns: not significant; **p < 0.01; ***p < 0.001; ****p < 0.0001).

Using the successfully established pulmonary metastatic melanoma mouse model to evaluate the in vivo synergistic anti‐tumor effects of Ce6‐pTP‐CsA. The treatment regimen is outlined in **Figure** **7**a. Figure 7b illustrates the inhibitory effects of different formulations on pulmonary metastatic melanoma. Quantification of surface metastatic nodules in the lungs was performed to determine the metastasis inhibition rates. The results revealed that the TP, pTP, Ce6‐CsA, pTP‐CsA, and Ce6‐pTP‐CsA groups achieved metastasis inhibition rates of 11.17 ± 10.96%, 21.51 ± 6.42%, 32.89 ± 13.43%, 56.83 ± 10.05%, and 88.83 ± 1.80%, respectively (Figure 7c,d; Table S3, Supporting Information). The mice in the Ce6‐pTP‐CsA group exhibited sparse yellow fluorescence from tumor metastases on the lung surface, indicating a significant tumor suppression effect. When compared to groups where CsA carried a single drug, the combination of Ce6 and pTP demonstrated superior therapeutic efficacy, achieving synergistic effects against metastatic melanoma.

Histological analysis using H&E staining was conducted to assess tumor tissue area proportions in lung sections across treatment groups, which were determined to be 11.96 ± 4.52%, 10.37 ± 2.25%, 6.66 ± 1.5%, 4.17 ± 0.95%, 3.59 ± 0.56%, and 2.02 ± 0.64%, respectively (Figure 7e). Among all groups, Ce6‐pTP‐CsA treatment resulted in the lowest tumor tissue area proportion. Although the Ce6‐CsA group showed significant inhibition of superficial melanoma nodules in the lungs, its suppression of deeper tumor nodules was relatively weak, potentially due to the limited penetration of the laser.^[^ ^48^, ^49^ ^]^ The positive rate of Ki67 reflects the proliferation activity of the tumor tissue, while the recurrence and metastasis of tumors are closely related to Ki67 levels.^[^ ^50^, ^51^ ^]^ Analysis revealed that the Ce6‐pTP‐CsA group exhibited the lowest Ki67 positive rate of 34.96 ± 5.33%, indicating that this combination of PDT and natural active ingredients effectively suppressed lung tumor proliferation (Figure 7f).

### 2.8 Biosafety and Biocompatibility

Considering the hepatotoxicity and nephrotoxicity associated with TP in clinical treatment, the biological safety of Ce6‐pTP‐CsA was assessed through body weight changes, organ H&E pathological sections, blood routine tests, and biochemical indicators in SCID mice. As shown in Figure S16 (Supporting Information), all groups demonstrated a gradual increase in body weight, except for the TP group, suggesting that TP may induce hepatic injury in SCID mice, potentially resulting in metabolic dysfunction. H&E staining results of major organs revealed inflammatory cell infiltration in the renal medulla and cortex in the TP group, disordered hepatic cell arrangement, and some signs of inflammatory and congestive dilation in the hepatic sinusoids, whereas Ce6‐pTP‐CsA groups showed no abnormalities in any organ sections (Figure S17, Supporting Information). The liver serves as the largest reticuloendothelial phagocytic system in the human body, yet the passive targeting ability of Ce6‐pTP‐CsA promotes greater drug distribution in the lungs, reducing hepatotoxicity through enzyme‐related metabolic decomposition in tumor tissues and lungs.^[^ ^52^, ^53^ ^]^

Additionally, hematological and biochemical analyses (Tables S4 and S5, Supporting Information) demonstrated that TP treatment significantly elevated inflammatory cell counts, including white blood cells (WBC), lymphocytes, and neutrophils, along with increased levels of alanine aminotransferase (ALT) and aspartate aminotransferase (AST). In contrast, Ce6‐pTP‐CsA treated groups showed no significant difference compared to the control group. These results indicate that TP induces tissue and hepatic oxidative stress via ROS production, culminating in lipid peroxidation and DNA damage. Notably, the Ce6‐CsA, pTP‐CsA, and Ce6‐pTP‐CsA groups did not exhibit significant changes in the counts of white blood cells, granulocytes, or lymphocytes, indicating that CsA‐based formulations showed no signs of triggering immune responses. Besides, the low immunogenicity of CsA helps prevent excessive cellular stress, and the sustained release characteristics of Ce6‐pTP‐CsA can further mitigate the inflammatory response induced by TP.^[^ ^53^ ^]^ Moreover, CsA's role as a drug delivery carrier reduces hepatic TP distribution, thereby attenuating hepatotoxicity. Collectively, these results demonstrate that Ce6‐pTP‐CsA exhibits excellent biological safety, and the combination of CsA, pTP, and Ce6 achieves enhanced efficacy with reduced toxicity, representing a promising therapeutic strategy for metastatic melanoma treatment.

## 3 Conclusion

In conclusion, this study presents a reverse thinking approach based on tumor tissue characteristics, specifically focusing on adipocyte recruitment, degradation and their capacity for fatty acids extensive uptake. We developed cryoshocked adipocytes, rendered non‐viable through liquid nitrogen treatment, as an innovative delivery platform for encapsulating the lipophilic prodrug pTP and photosensitizer Ce6 in the context of pulmonary metastatic melanoma treatment. The implementation of CsA effectively overcomes the limitations of conventional adipocytes, including low drug‐loading capacity and poor systemic biocompatibility, while simultaneously achieving targeted pulmonary drug delivery. Furthermore, pTP and Ce6 act synergistically to inhibit tumor cell invasion and migration through the activation of endogenous mitochondrial apoptosis and endoplasmic reticulum stress pathways, ultimately promoting tumor cell apoptosis. Of particular significance, the disulfide bond cleavage in pTP results in GSH consumption, thereby reducing GSH‐mediated ROS scavenging and consequently enhancing photodynamic therapeutic efficacy. Moreover, the trilateral combination of Ce6, pTP, and CsA demonstrated robust inhibition of tumor proliferation in a mouse model of melanoma lung metastasis. To summarize, this research establishes a novel biomimetic drug delivery strategy for metastatic melanoma treatment, successfully integrating natural active ingredients with PDT, thus offering innovative perspectives for antitumor formulation development and potential future clinical applications.

## 4 Experimental Section

### Materials

Triptolide and Ce6 were purchased from Shanghai Yuanye Biotechnology Co., Ltd (Shanghai, China). Dexamethasone (DXMS) and 3‐Isobutyl‐1‐methylxanthine (IBMX) were purchased from Sigma–Aldrich (USA). Recombinant Human Insulin was supplied by Novo Nordisk (Denmark). Dulbecco's modified Eagle medium (DMEM), trypsin‐EDTA (0.25%), fetal bovine serum (FBS) for A375‐M1 cell, and penicillin–streptomycin (PS) were purchased from Gibco Life Technologies (NY, USA). FBS for 3T3‐L1 was purchased from Zhejiang Tianhang Biotechnology Co., Ltd (Hangzhou, China). CCK‐8 kit, Oil Red‐O stain‐ing kit and Ki67 kit were obtained from Solarbio Life Science Co., Ltd (Beijing, China). BODIPY 505/515 and ROS assay kit (DCFH‐DA) were purchased from MedChemExpress (USA). V‐FITC/PI apoptosis kit and GSH were purchased from Solarbio (Beijing, China). Biotech CE Dialysis Tube MD31 (MWCO: 2000 kDa) was purchased from Spectrumlabs (USA), transwell cell culture chamber was purchased from Falcon (USA), Hoechst 33342 was purchased from Beyotime Biotechnology (hanghai, China), DPBF was purchased from MedChemExpress (USA), Bouins The fixative was purchased from Sbjbio‐Z (Nanjing, China), and fluoro[18]deoxyglucose injection (18F‐FDG) was a gift from the Department of Nuclear Medicine, Fudan University Cancer Hospital. The antibodies used for Western blotting, including FATP1, FABP4, CD36, CHOP, and ATF4, were obtained from Proteintech (IL, USA); P‐elF2*α* and Bcl‐2 primary antibodies were from Abclonal (MA, USA); the PERK primary antibody was from Servicebio (Wuhan, China); and the Cleaved PARP, Cleaved‐Caspase 3, and Cleaved‐Caspase 9 primary antibodies were from CST (MA, USA).

### Cell Lines and Animals

A375‐M1 was purchased from ATCC Cell Bank (USA), and NOD SCID mice were from Beijing Vital River Laboratory Animal Technology Co., Ltd. (Beijing, China). All animal experiments were conducted in accordance with the 3Rs principle and received approval from the Ethics Committee of Fudan University Shanghai Cancer Center, under the approval number 2020 FUSCC JS‐145.

### Induction and Identification of Adipocyte Differentiation

Mouse fibroblasts (3T3‐L1) in the logarithmic phase were dissociated using trypsin and seeded at a density of 5 × 10^5^ cells per well in a 6‐well plate. After contacting inhibition for 24 h, add 2 mL of Induction Solution I to each well and incubate for 48 h. Then replace with fresh Induction Solution I and continue culturing for another 48 h. Subsequently, induction medium II (a complete medium containing 10 µg·mL^−1^ insulin) was used for an additional 48 h of incubation. Finally, the cells were cultured in a normal medium for 48–96 h to yield mature, lipid‐filled adipocytes. Lipid accumulation was assessed using Oil Red O staining and observed under a microscope.

### Preparation and Activity Assay of CsA

Apo cells induced to mature in the same batch were digested with trypsin. A cryopreservation solution consisting of a complete medium with 1% DMSO was mixed at a ratio of 1 mL to 2 × 10^6^ Apo cells and placed into cryovials, then immersed in liquid nitrogen for 12 h to prepare blank CsA carriers.

Both Apo cells and CsA were thawed at 37 °C. The cells were resuspended in a complete medium to adjust the concentration to 2 × 10^5^ cells·mL^−1^. Next, 100 µL of the cell suspension was added to each well of a 96‐well plate for incubation. On days 1, 2, 3, and 4, 10 µL of CCK‐8 reagent was added to each well and incubated for 1.5 h. Absorbance at 450 nm (n = 6) was measured to determine the viability of Apo cells and CsA.

### Preparation of Ce6‐pTP‐CsA

CsA was taken out of liquid nitrogen and thawed. After adding five times the volume of PBS, the suspension was centrifuged at 1000 rpm for 1 min, and the supernatant was discarded. A drug‐loading PBS solution containing both Ce6 (50 µg·mL^−1^) and pTP (500 µg·mL^−1^) was prepared. The drug‐loading solution and CsA were mixed at a ratio of 1 mL to 2 × 10⁶ cells and incubated at 40 °C, protected from light, for 2 h. After incubation, the solution was cold‐shrunk at 4 °C for 10 min, followed by resuspension in pre‐chilled PBS and centrifugation at 1000 rpm·min^−1^. The supernatant was discarded, and Ce6‐pTP‐CsA was obtained. The methods for preparing Ce6‐CsA and pTP‐CsA followed the same procedure as Ce6‐pTP‐CsA.

### Characterization of Ce6‐pTP‐CsA

For morphological observation, Ce6‐pTP‐CsA was washed with PBS and fixed at room temperature in glutaraldehyde solution for 2 h. The fixed sample was washed with 0.1 M phosphate buffer (PBS, pH 7.4). Next, 1% osmium acid prepared in PBS was used for light‐protected fixation at room temperature for 1–2 h, followed by additional PB washing. The cells were dehydrated using graded ethanol, with the final dehydration step involving isoamyl acetate for 15 min. The samples were dried and attached to double‐sided carbon conductive tape before being placed in a sputter coater for gold spraying for ≈30 s. The working voltage was set to 3.0 kV, and the samples were observed under a scanning electron microscope for image capture.

For particle size distribution, Ce6‐pTP‐CsA was diluted to a concentration of 5 × 10⁶ cells·mL^−1^. Samples were prepared for observation, and the particle size was statistically analyzed from 7 randomly selected fields under a bright‐field fluorescence inverted microscope. The particle size distribution was plotted, and the data were fitted using a Gaussian model.

The Total protein of Ce6‐pTP‐CsA was identified. Apo cells, CsA, and Ce6‐pTP‐CsA (2 × 10⁶ cells each) were dried, and the residual liquid was removed. RIPA lysis buffer was added to the cells on ice. The samples were centrifuged at 120 00 rpm at 4 °C, and the supernatant was collected as the total protein solution. The samples were run on SDS‐PAGE following the preparation of separating gels. After electrophoresis, the gels were stained with Coomassie Brilliant Blue at 70 °C for 5 min. After washing the gels with water, the gels were de‐stained, and photographs of the target protein bands were taken.

### In Vitro Drug Release Study

The release characteristics of Ce6‐pTP‐CsA were examined under pH 7.4 and pH 5.0 conditions. Dialysis bags were cut into small segments and activated with ultrapure water. Each bag was loaded with Ce6‐pTP‐CsA PBS suspension (3 × 10⁶ cells·mL^−1^, 0.5 mL). The dissolution conditions were set at 37 °C and 100 rpm min^−1^ in PBS (200 mL) with 10% ethanol. At predetermined time points, the dialysis bags were removed, and the contents were centrifuged to collect the precipitate. The release of pTP and Ce6 was measured by HPLC and UV spectroscopy.

### Cell Viability Assay

The cytotoxicity of Ce6‐pTP‐CsA was evaluated by CCK‐8 assay. A375‐M1 cells were seeded into 96‐well plates, and when confluence was reached, TP, pTP, Ce6, Ce6‐pTP, pTP‐CsA, Ce6‐pTP‐CsA, and DMEM were added separately. After 24 h of incubation, the cells were irradiated with a laser generator at 660 nm wavelength and 50 mW power for 2 min, followed by another 24 h of incubation. Then, 10 µL of CCK‐8 reagent was added to each well, and the absorbance was measured at 450 nm to calculate cell viability. GraphPad was used to fit the log concentration/viability curve and calculate the IC50 values (n = 6).

### Invasion Assay

A375‐M1 cells were treated with TP, pTP, Ce6‐CsA, pTP‐CsA, and Ce6‐pTP‐CsA (50 nmol·L^−1^ TP) for 24 h, followed by irradiation at 660 nm wavelength and 50 mW power for 2 min and subsequent incubation for 24 h. The treated cells were added to the upper chamber of a transwell insert pre‐coated with Matrigel (Falcon, China) and placed in a 24‐well plate containing 600 µL of a complete medium in the lower chamber. After 24 h, non‐invading cells were removed, the inserts were fixed with 4% paraformaldehyde, stained with 1% crystal violet, and photographed under an inverted microscope (n = 5).

### Migration Assay

A375‐M1 cells in the logarithmic growth phase were seeded in 6‐well plates. Upon confluence, a 200 µL pipette tip was used to create a scratch in each well. Cells were treated with DMEM containing 2% fetal bovine serum, TP, pTP, Ce6‐CsA, pTP‐CsA, and Ce6‐pTP‐CsA (50 nmol·L^−1^ TP) for 24 h. After laser irradiation, the cells were cultured for another 24 h. Photographs were taken at 0 and 36 h under a fluorescence microscope, and the wound area was quantified using Image J (n = 3).

### Apoptosis Assay

The cells were treated similarly to the Migration Assay. After treatment, the cells were washed with pre‐chilled PBS and centrifuged to collect the cell pellet. Annexin V/FITC was added to the cell suspension and incubated at room temperature, protected from light, for 5 min, followed by the addition of 5 µL of propidium iodide (PI) and 400 µL of PBS. Apoptosis and necrosis were analyzed by FCM (BD, USA).

### pTP Transformation in Tumor Microenvironment In Vitro

A methanol‐PBS (1:1) solution of 1000 nmol pTP (10 mL) was prepared. Reactions were performed in 15 mL EP tubes under the following conditions: 10 mM GSH (pH = 5.0), 1 µM GSH (pH = 7.4), and 1 µM GSH (pH = 7.4), with stirring at 100 rpm at 37 °C. Samples were taken at predetermined time points, and pTP conversion was analyzed by HPLC. Fresh conversion media were replenished after sampling.

### Investigation of the Interaction Between A375‐M1 Cells and Ce6‐pTP‐CsA

A375‐M1 cell suspensions were seeded onto 24‐well plates containing coverslips. Once confluence was reached, Ce6‐pTP‐CsA labeled with BODIPY 505/515 was added. After 12, 24, and 48 h of incubation, the cells were fixed with 4% paraformaldehyde at room temperature, stained with Hoechst 33342 to label nuclei, and washed with PBS before being mounted with an antifade mounting medium. The uptake and degradation of Ce6‐pTP‐CsA were observed using a CLSM (LSM800, ZEISS, Germany) with 505/515 nm Ex/Em for BODIPY and 350/460 nm Ex/Em for nuclear localization.

A375M1 cells were seeded onto 24‐well plates containing coverslips, with 600 µL of culture medium added to each well. A Transwell chamber (Falcon, China) coated with Matrigel was placed in each well. For the control group, Ce6‐pTP‐CsA was added to the upper chamber of the Transwell (separated from the A375M1 cells). For the experimental group, Ce6‐pTP‐CsA was added directly to the lower chamber (in direct contact with the A375M1 cells). After incubation for 3, 12, and 24 h, the cells were fixed at room temperature with 4% paraformaldehyde. DAPI was then added to stain the nuclei. Following staining, the cells were washed with PBS and mounted using an anti‐fade mounting medium. The uptake of Ce6 by A375M1 cells was observed using a laser confocal microscope (400/660 nm Ex/Em).

A375‐M1 cells were treated with pTP, PA, and Ce6‐pTP‐CsA for 24 h, followed by laser irradiation and 12 h of additional incubation. Cells were collected using a cell scraper, lysed on ice, and protein concentration was determined using a BCA assay. Samples were subjected to SDS‐PAGE (70 V, constant voltage). Blots were incubated with anti‐FATP1 (1:1000), anti‐FABP4 (1:1000), and anti‐CD36 (1:1000) primary antibodies at 4 °C overnight, followed by HRP‐labeled goat anti‐rabbit secondary antibody (1:2000) or HRP‐labeled goat anti‐mouse secondary antibody (1:2000) at room temperature for 2 h. Chemiluminescence detection was performed using an automated chemiluminescence imaging system (Tanon 5200, China), and gray value analysis was performed using the TanonImage system (n = 3).

### Investigation of the Uptake Mechanism of Ce6‐pTP‐CsA

BEAS‐2B cells and RAW264.7 cells were seeded onto 24‐well plates containing coverslips. After reaching confluence, BODIPY 505/515‐labeled Ce6‐pTP‐CsA was added to the wells. Following incubation for 12, 24, and 48 h, the cells were fixed at room temperature using 4% paraformaldehyde. DAPI was then used to stain the nuclei, and the cells were washed with PBS before being mounted with an anti‐fade mounting medium. The uptake and distribution of the formulation were observed using a laser confocal microscope (LSM900, ZEISS, Germany) under 505/515 nm excitation/emission conditions.

### Mechanism of Ce6‐pTP‐CsA Photodynamic Action

A375‐M1 cells were seeded into well plates and treated with Ce6‐pTP‐CsA in a complete medium. After incubation under dark conditions, Ce6 uptake by A375‐M1 cells was observed using a CLSM (Ex/Em 400/660 nm) and FCM.

A375‐M1 cells were treated with TP, pTP, Ce6‐CsA, pTP‐CsA, and Ce6‐pTP‐CsA (50 nmol·L^−1^ TP) for 24 h, followed by incubation with a complete medium containing 5 µM H2DCFDA for 30 min at 37 °C, protected from light. The cells were then irradiated with a laser and incubated for an additional 6 h. After fixing with 4% paraformaldehyde and mounting, fluorescence intensity was observed using a CLSM (Ex/Em = 488/525 nm). For ROS detection, A375‐M1 cells were treated with different formulations and incubated with 10 µM DPBF in a complete medium for 40 min. After laser irradiation and 30 min of incubation, the cells were digested with trypsin, and MFI was measured by FCM.

Western blot analysis of the endoplasmic reticulum pathway followed the same procedure described earlier. Ce6‐pTP‐CsA (+) indicates laser irradiation after 24 h of drug treatment, while Ce6‐pTP‐CsA (−) indicates no irradiation. The primary antibodies used were anti‐GRP78 (1:1000), anti‐ATF4 (1:1000), anti‐CHOP (1:1000), anti‐P‐eIF2*α* (1:1000), anti‐PERK (1:1000), and anti‐Cleaved‐Caspase 12.

### Western Blot Analysis of Combined Mechanisms

The Western Blot procedure followed the aforementioned protocol. The primary antibodies used were anti‐Bax (1:1000), anti‐Bid (1:1000), anti‐cleaved PARP (1:1000), anti‐Cleaved‐Caspase 3 (1:1000), anti‐Bcl 2 (1:1000), anti‐Cleaved‐Caspase 9 (1:1000), and anti‐Cytochrome C (1:1000).

### Biodistribution

A pulmonary metastasis model was successfully established in SCID mice via tail vein injection of A375‐M1 cells. A DiR staining solution (1 mg·mL^−1^) was prepared, and 1 mL of this solution was used to label 1 × 10⁷ Ce6‐pTP‐CsA cells. The model mice (n = 3) were randomly divided into two groups and intravenously injected with DiR or Ce6‐pTP‐CsA (1 × 10⁶ cells per 20 g). At predetermined time points, the mice were anesthetized, and whole‐body fluorescence intensity distribution images were captured using a small animal in vivo optical imaging system (PerkinElmer IVIS, USA). After 12 h, the mice were euthanized, and the heart, liver, spleen, lungs, kidneys, and blood were collected for fluorescence intensity imaging.

The model mice were randomly divided into three groups and injected with TP, pTP, or Ce6‐pTP‐CsA. At predetermined time points, blood was collected from the orbital cavity and centrifuged to obtain plasma. The mice were euthanized at different times, and their hearts, livers, spleens, lungs, and kidneys were weighed and homogenized. A 20 µL solution of carmatine internal standard (100 ng·mL^−1^ in methanol) was added to the homogenates, followed by the addition of 880 µL methanol. After vortexing, the samples were centrifuged at 15000 rpm at 4 °C. The supernatant was collected, dried in a vacuum concentrator, and reconstituted with methanol. After centrifugation, 10 µL of the sample was injected for HPLC‐MS analysis. Plasma samples were processed similarly to tissue samples.

### Antitumor Efficacy In Vivo

A pulmonary metastasis model was established in SCID mice as described above. Ten days after model establishment, the mice were randomly divided into five groups and intravenously injected with PBS, TP, pTP, Ce6‐CsA, pTP‐CsA, or Ce6‐pTP‐CsA every other day for a total of six injections. The dosing was calculated based on a TP concentration of 0.6 mg·kg^−1^. Twelve hours after the last injection, the mice were anesthetized with isoflurane, and their chest areas were shaved. Each mouse received laser irradiation for 5 min. During the treatment period, the mice's body weight was recorded, and the mice were euthanized 2 weeks after the start of treatment. The lungs were collected, stained with Bouin's fixative, and the number of surface nodules was counted. The metastasis index and metastasis inhibition rate were calculated (n = 5). The metastasis inhibition rate was calculated as (1‐number of nodules in the experimental group / average number of nodules in the control group) × 100%.

H&E‐stained lung sections from each group of SCID mice were prepared, and the melanoma lung metastasis and tumor area relative to total lung area were assessed (n = 3). Ki67 staining was performed to evaluate tumor proliferation. The main steps included deparaffinization, antigen retrieval, hydration, serum blocking, incubation with anti‐Ki67 antibody (1:300), incubation with HRP‐labeled goat anti‐mouse secondary antibody (1:200), DAB staining, and microscopic examination (Aperio Technologies, USA).

### Biosafety Evaluation

Fresh red blood cells (RBCs) were collected from SCID mice and centrifuged at 4000 rpm. The RBCs were washed with saline to obtain pure RBCs. PBS and 1% Triton X‐100 were used as negative and positive controls, respectively. Hemoglobin leakage was detected by measuring the OD at 540 nm. The hemolysis rate (%) was calculated as (sample absorbance – average absorbance of the negative control)/ (average absorbance of the positive control – average absorbance of the negative control) × 100%.

Mice's body weight was monitored after the first injection to assess the safety of the formulation. After the efficacy study, the heart, liver, spleen, and kidneys of each group of SCID mice were collected and analyzed for pathological changes using H&E staining. Blood samples were collected for complete blood count and biochemical analysis using an automated biochemistry analyzer (BC‐2800vet, Mindray, China).

### Statistical Analysis

Data were presented as mean ± SD. Statistical analysis was performed by GraphPad Prism. The T‐test was used for comparison between the two groups. One‐way ANOVA using the Tukey post‐test was applied for comparison among three or more groups.

## Conflict of Interest

The authors declare no conflict of interest.

## Supporting information



Supporting Information

## Data Availability

The data that support the findings of this study are available from the corresponding author upon reasonable request.
