# Supplementary material for: Cryoshocked Adipocytes Mediated Dual‐Modal Strategy Combining Photodynamic Therapy and Triptolide Palmitate for Pulmonary Metastatic Melanoma Treatment
Source: Adv Sci (Weinh). 2025 Jan 13;12(9):2414307. doi: 10.1002/advs.202414307 (PMC11884613; doi:10.1002/advs.202414307)
Supplement: Supplementary file 1 — Supporting Information [file ADVS-12-2414307-s001.docx]

**Supporting information**

**S1**


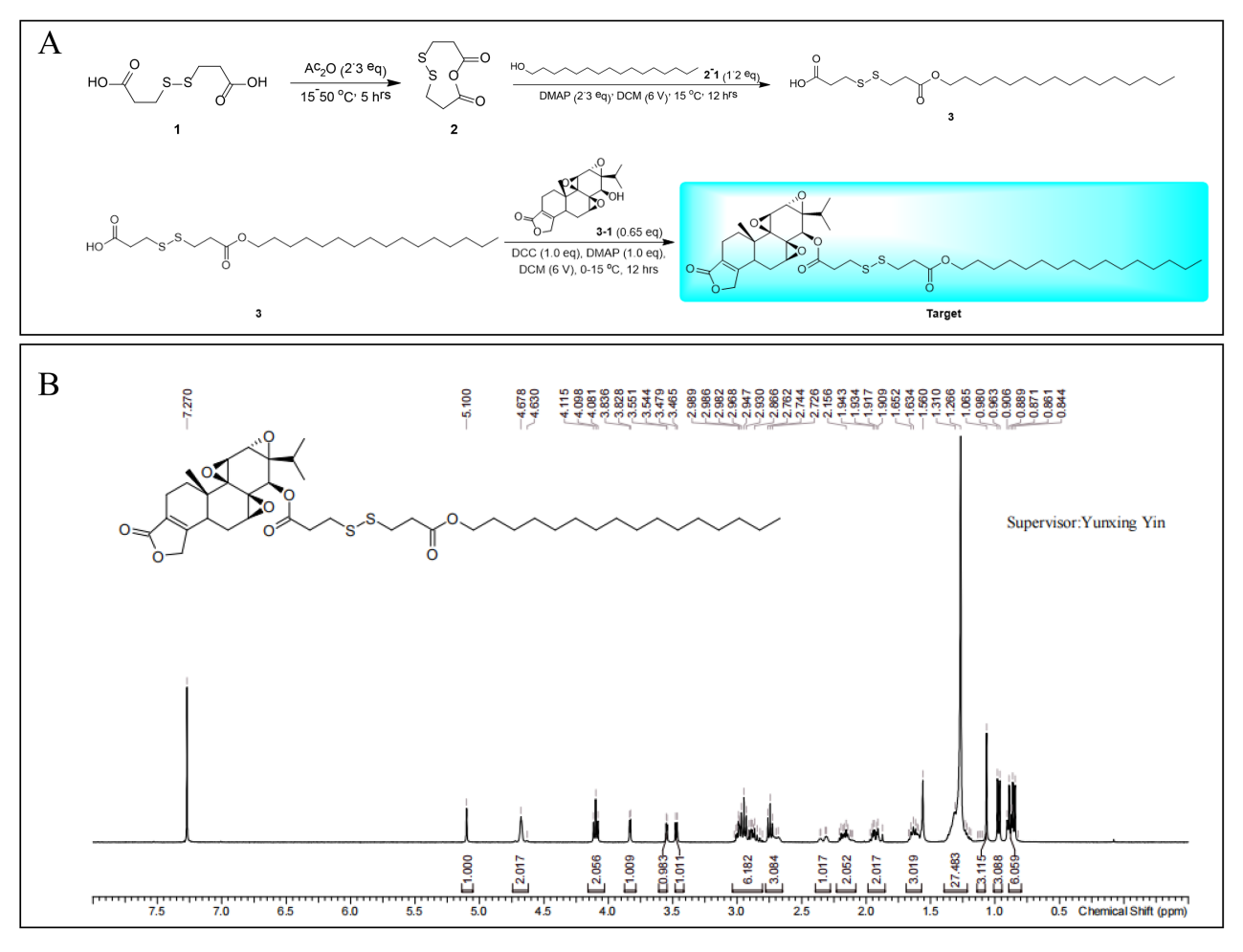


**Figure S1.** Synthesis and Characterization of pTP. A: Synthetic route of pTP; B: ^1^H NMR spectrum of pTP.

**S2**


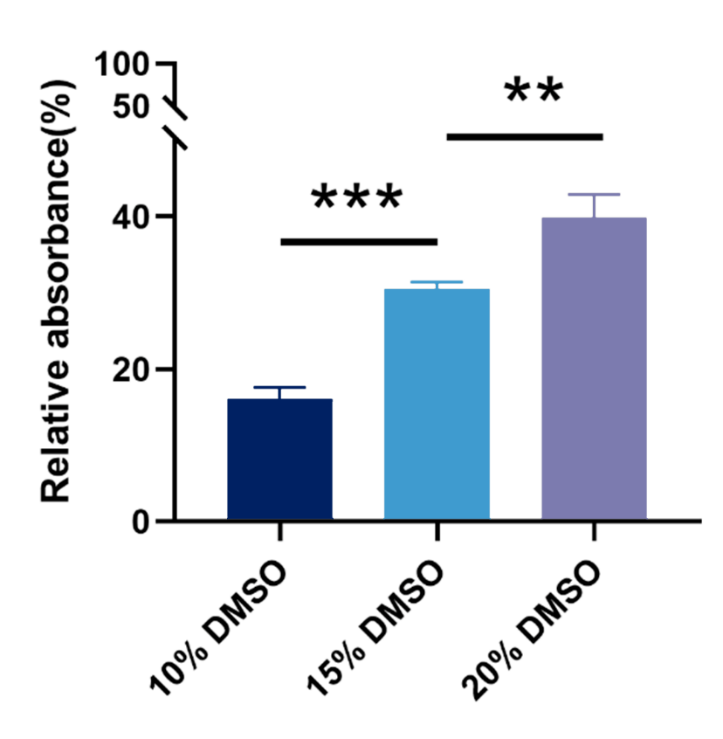


**Figure S2.** Effect of different proportions of DMSO on Apo activity (n=3). (**p < 0.01; ***p < 0.001)

S3


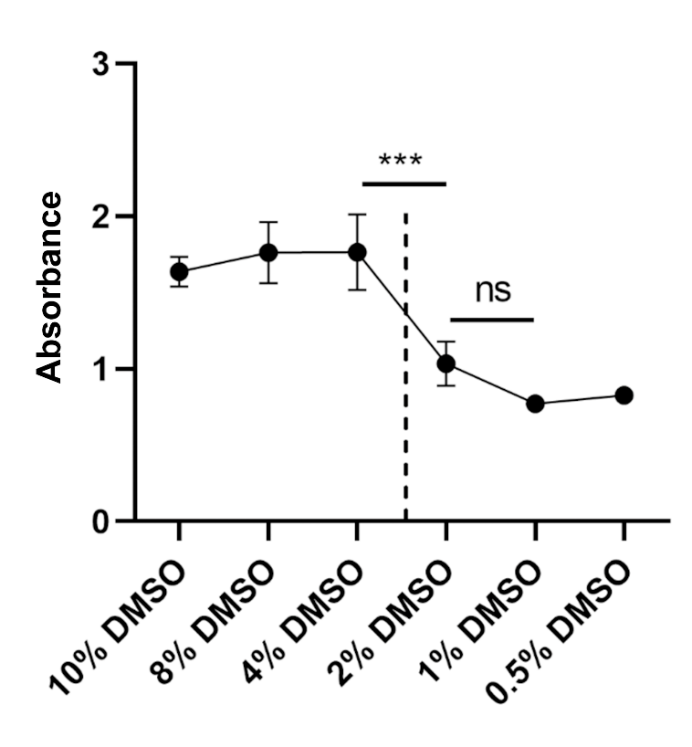


**Figure S3.** Effect of DMSO content in cryopreservation solution on Apo activity. ns: not significant (n=3). (ns, not significant; ***p < 0.001)

S4


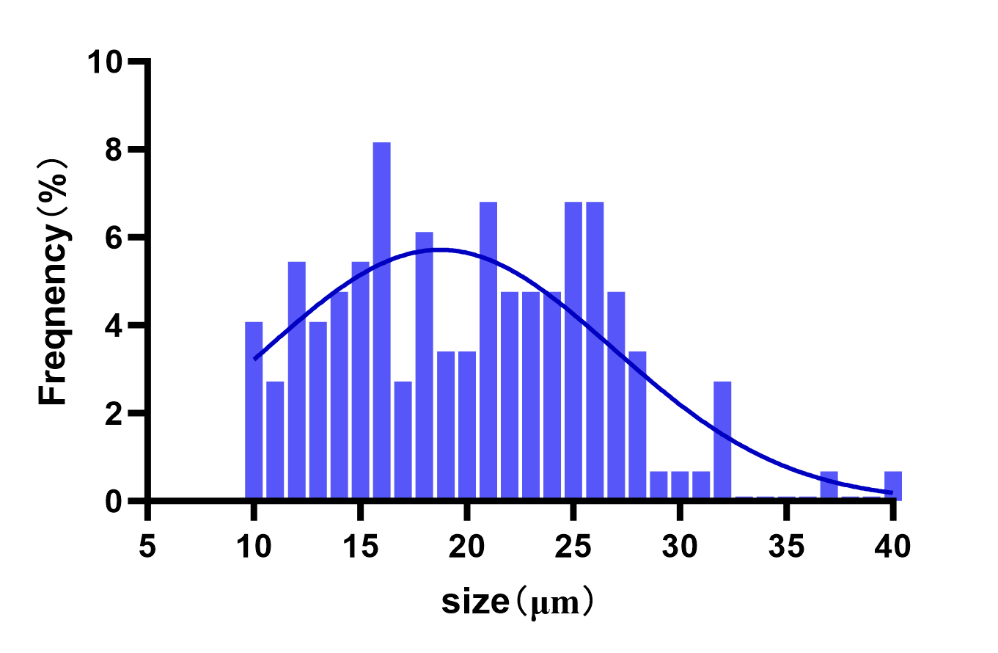


**Figure S4.** Particle size statistics of CsA carrier.

S5


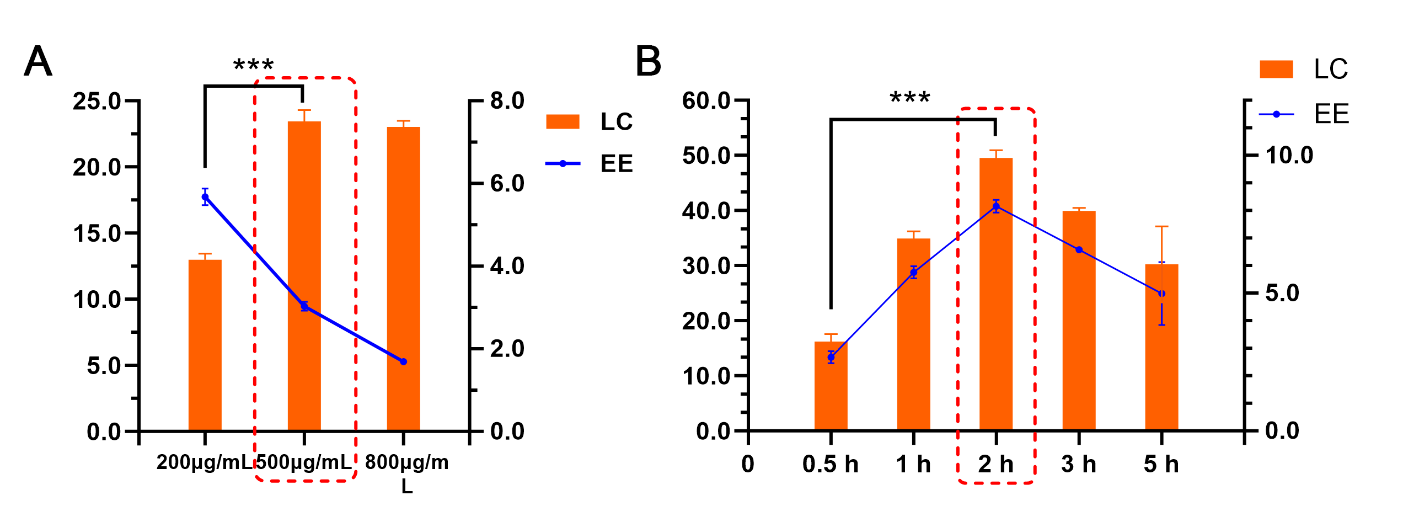


Figure S5. Investigation of pTP encapsulation efficiency and drug loading capacity. A: Drug loading concentration; B: Drug loading time (n=3). (***p < 0.001)

S6


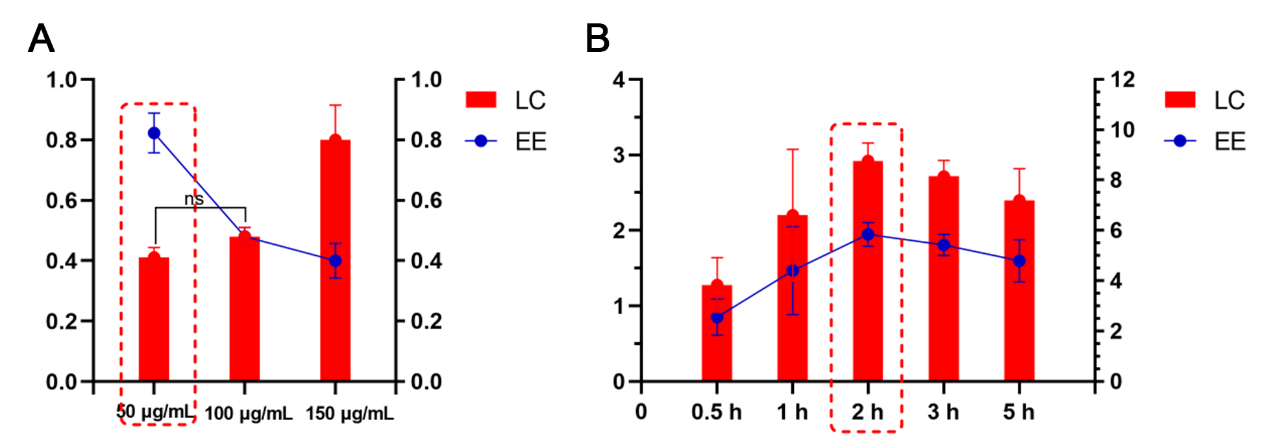


Figure S6. Investigation of Ce6 drug loading capacity and encapsulation efficiency. A: Drug loading concentration; B: Drug loading time (n=3). (ns, not significant)

S7


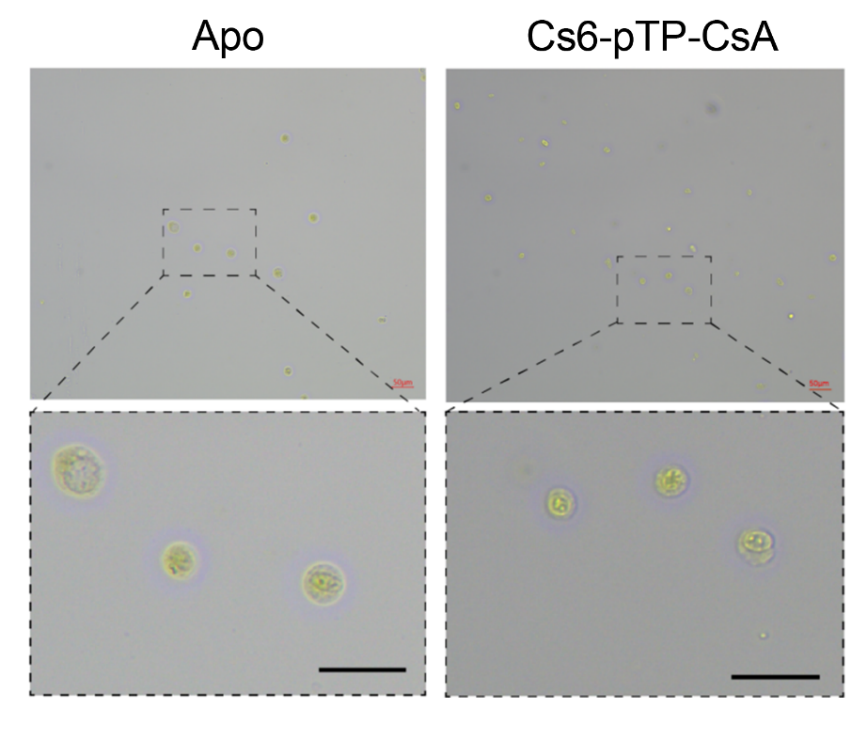


**Figure S7.** Bright-field images of Apo and Ce6-pTP-CsA under an inverted microscope. A: CsA; B: 5x magnification of a selected region in A; C: Ce6-pTP-CsA; D: 5x magnification of a selected region in C. Scale bar: 50 μm.

S8


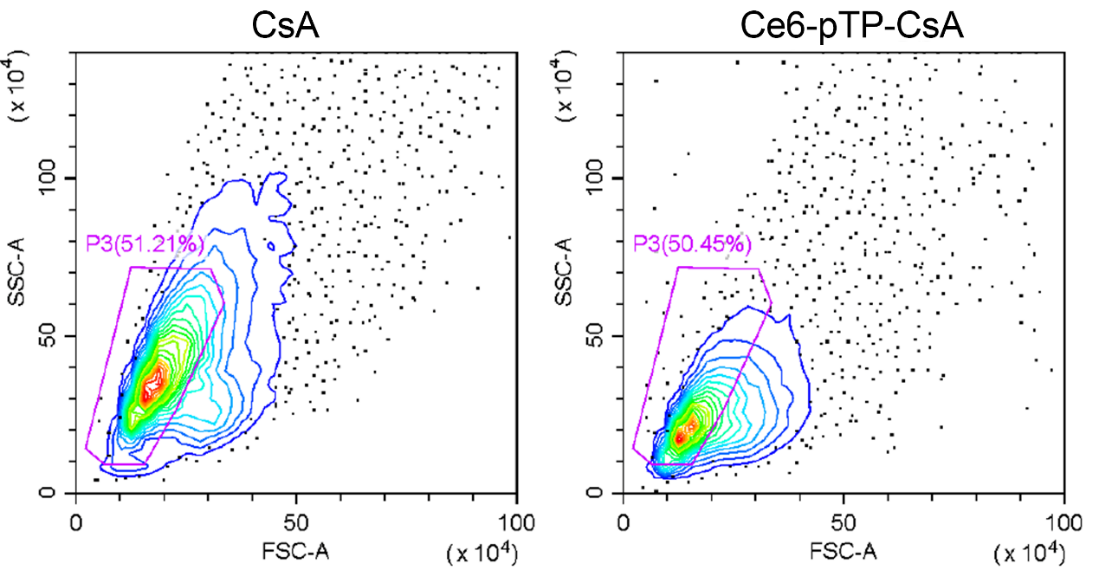


**Figure S8.** FCM analysis of forward scatter (FSC) and side scatter (SSC) for CsA and Ce6-pTP-CsA.

S9


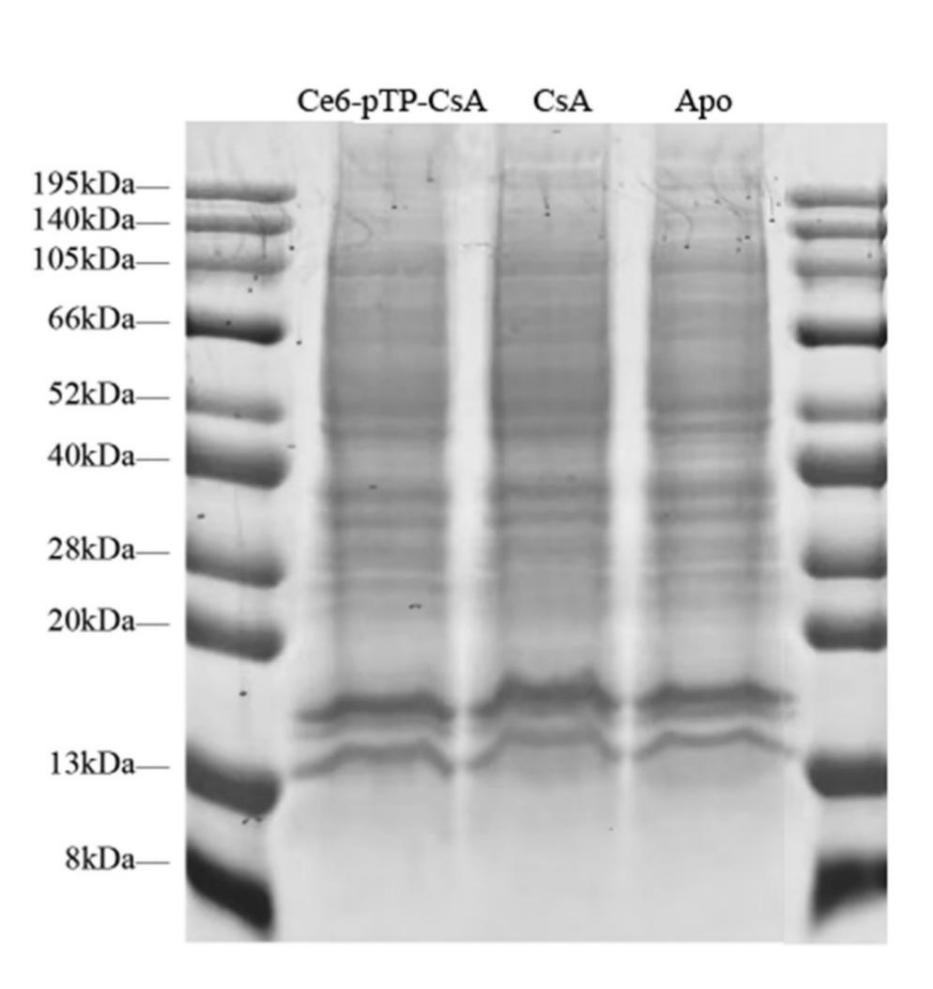


**Figure S9.** SDS-PAGE gel image for total protein identification.

S10


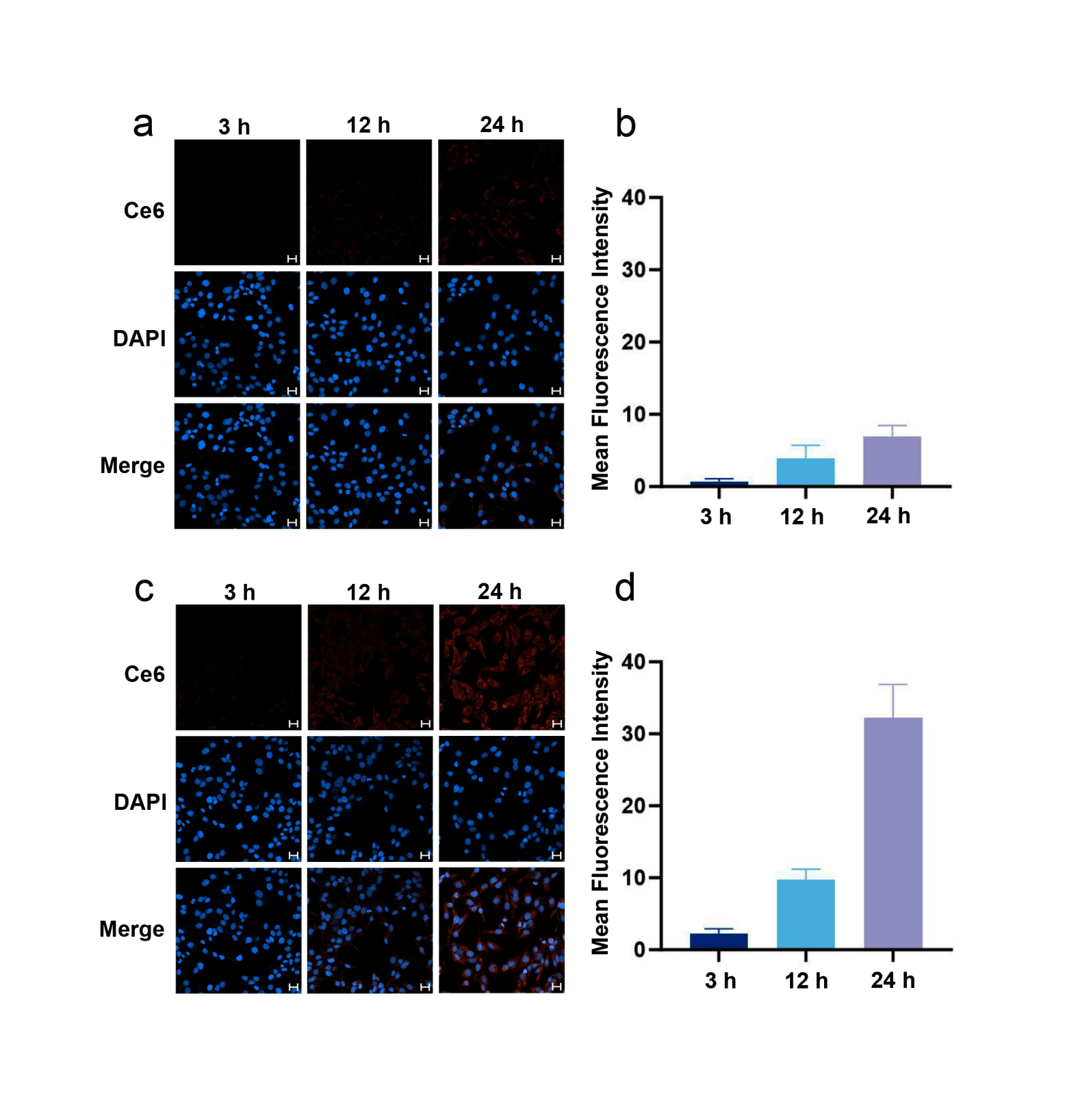


**Figure S10.** Cellular uptake of Ce6. a) Representative CLSM images and b) analysis of MFI showing the cellular uptake of Ce6-pTP-CsA added to the upper chamber of the Transwell and cultured separately from A375-M1 cells for 3 h, 12 h, and 24 h (n=3). Scale bar: 20 μm. c) Representative CLSM images and d) analysis of MFI showing the cellular uptake of Ce6-pTP-CsA added to the lower chamber of the Transwell and co-incubated with A375-M1 cells for 3 h, 12 h, and 24 h (n=3). Scale bar: 20 μm.

S11


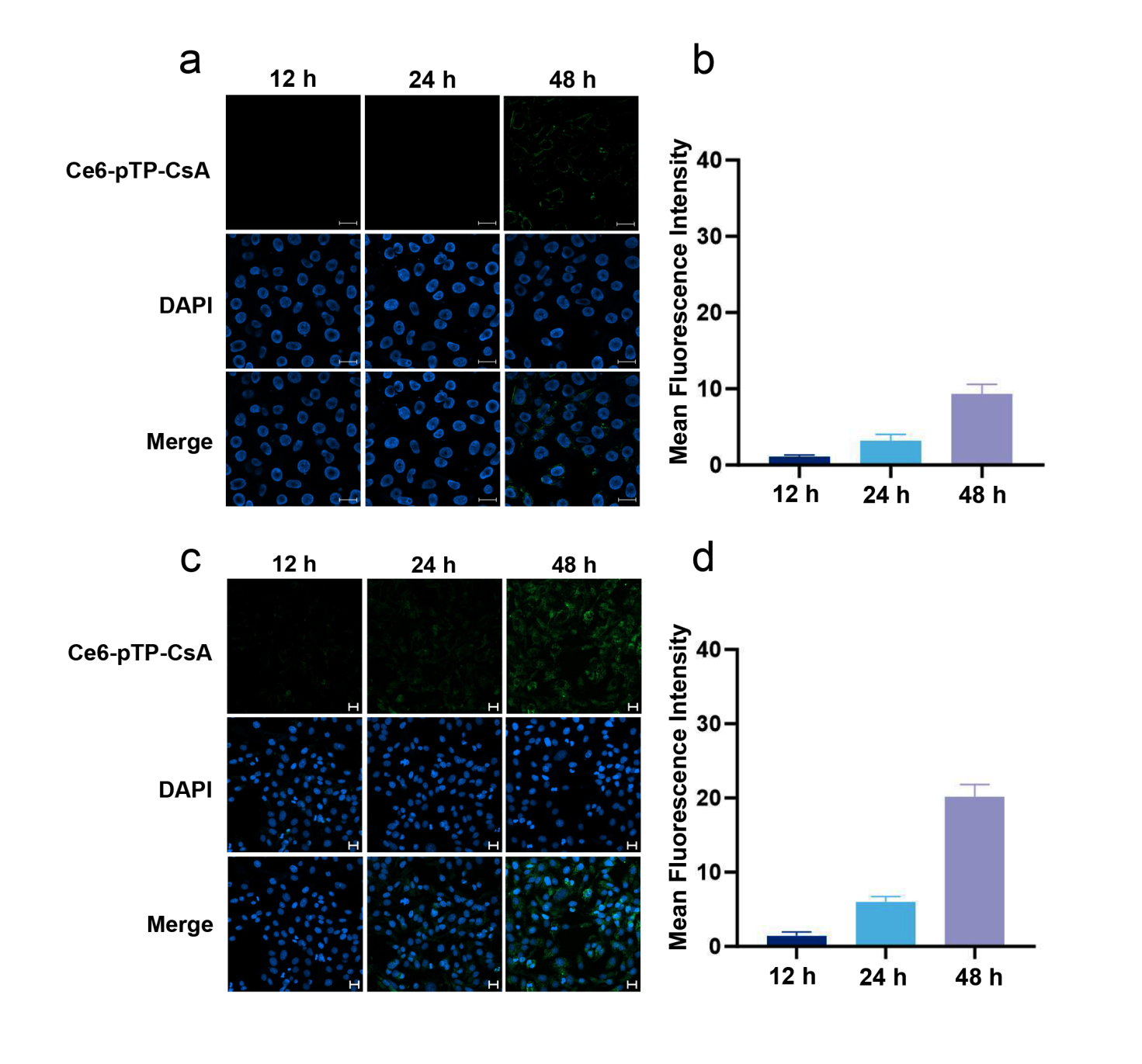


**Figure S11.** Uptake Mechanism of Ce6-pTP-CsA. a) Representative CLSM images and b) analysis of MFI showing the cellular uptake of Ce6-pTP-CsA co-incubated with BEAS-2B cells for 12 h, 24 h, and 48 h (n=3). Scale bar: 20 μm. c) Representative CLSM images and d) analysis of MFI showing the cellular uptake of Ce6-pTP-CsA co-incubated with RAW 264.7 cells for 12 h, 24 h, and 48 h (n=3). Scale bar: 20 μm.

S12


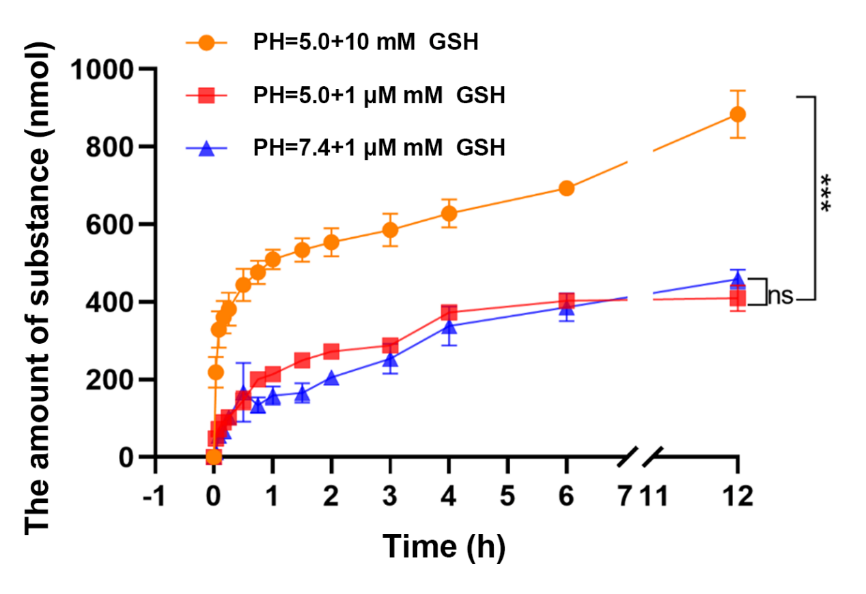


**Figure S12.** pTP in vitro drug transformation timeline in the tumor microenvironment (n=3). (ns, not significant; ***p < 0.001)

S13


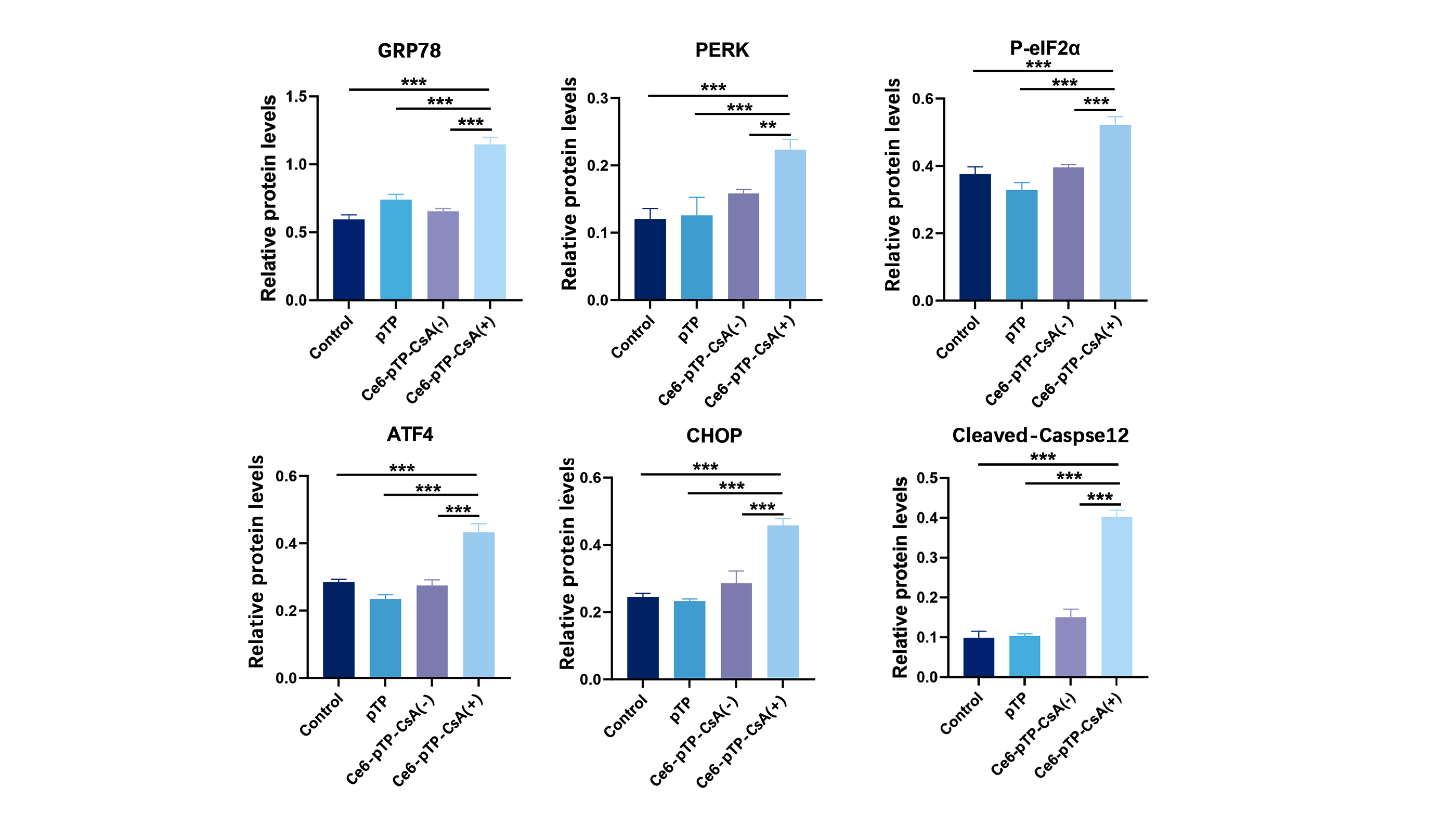


**Figure S13.** Western blot analysis of GRP78, PERK, P-eIF2α, ATF4, CHOP, and Cleaved-Caspase 12 expression in A375-M1 cells after treatment with PBS, pTP and Ce6-pTP-CsA (±hv) (n=3).

S14


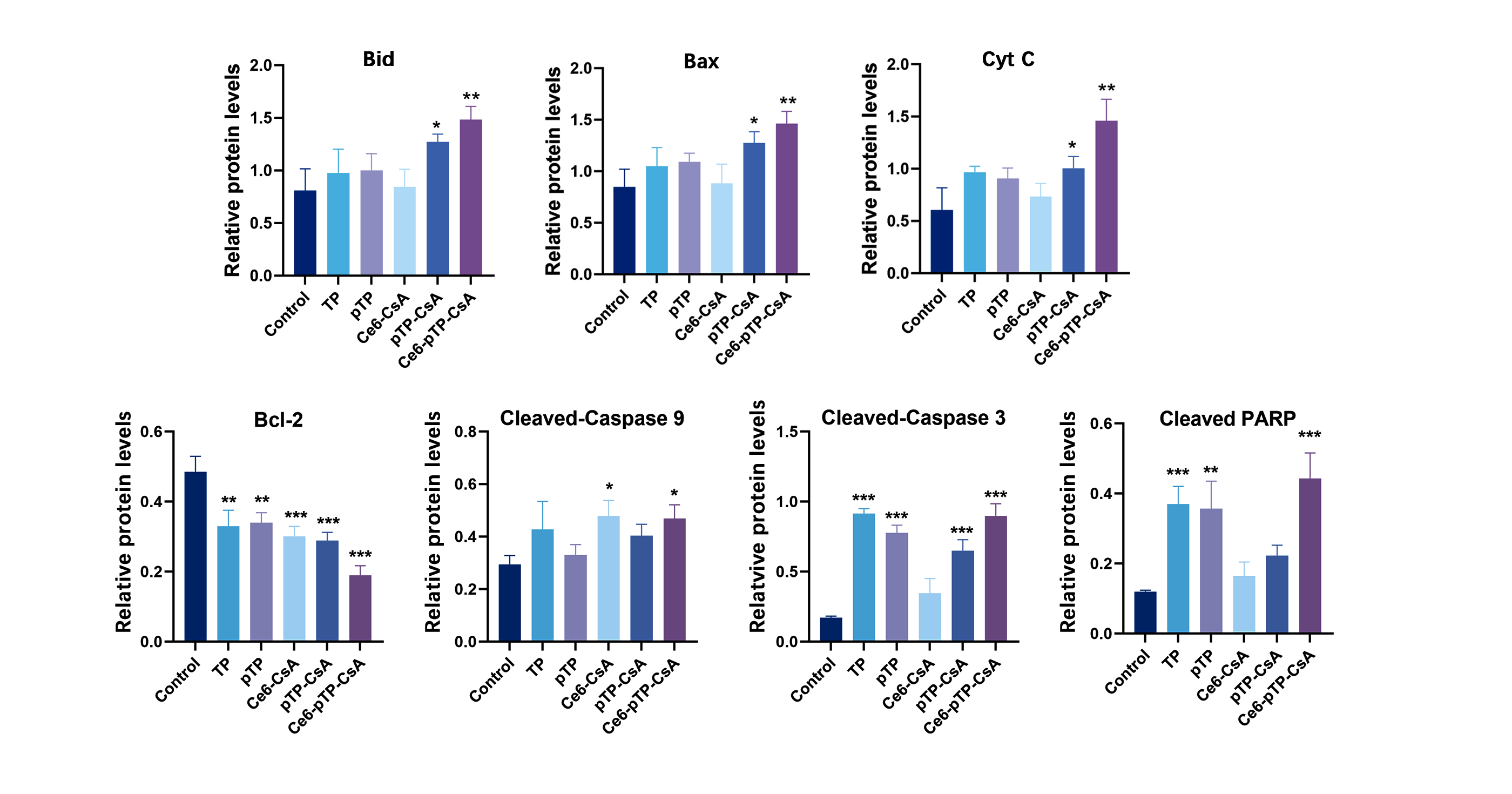


**Figure S14.** Western blot analysis of Bax, Bid, Cytochrome C, Bcl-2, Cleaved-Caspase 9, Cleaved-Caspase 3, and Cleaved PARP expression in A375-M1 cells after treatment with PBS, TP, pTP, Ce6-CsA, pTP-CsA, and Ce6-pTP-CsA (n=3).

S15


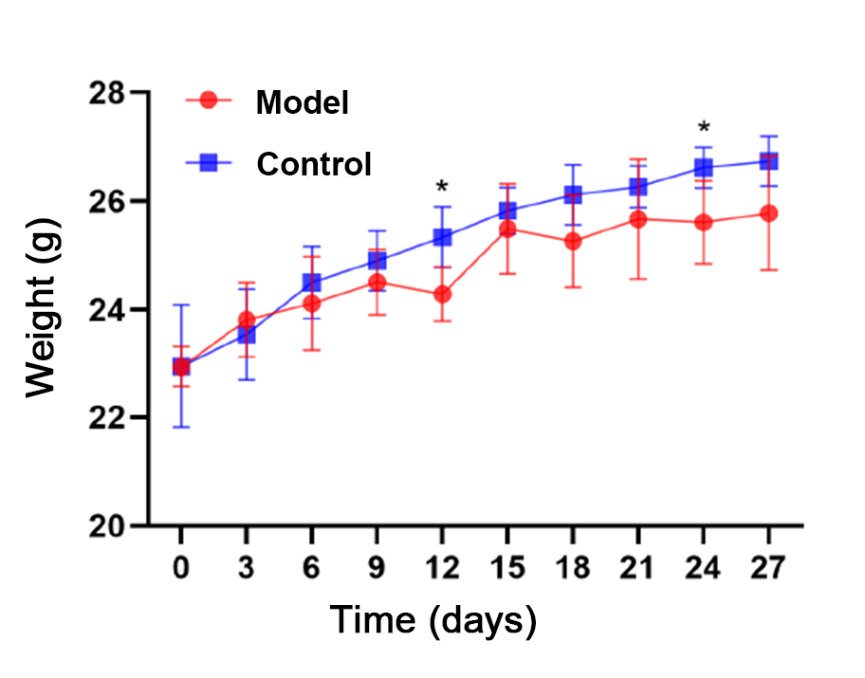


**Figure S15.** Line chart of body weight changes between the model group and the control group (n=6). (*p < 0.05)

Table S1

Pharmacokinetic Parameters of TP in the Lungs

| Group | | T_1/2_  (h) | C_max_  (μg L^-1^) | | | AUC_(0-t)_  (μg h L^-1^) | | MRT_(0-t)_  (h) |
| --- | --- | --- | --- | --- | --- | --- | --- | --- |
| TP | 0.264±0.121 | | | 197.555±30.251 | | | 98.322±30.565 | 0.295±0.033 |
| pTP | 0.429±0.132 | | | 305.338±50.565 | | 93.644±40.123 | | 0.324±0.052 |
| Ce6-pTP-CsA | 4.887±0.56 | | | 155.757±27.245 | 436.044±50.489 | | | 4.901±1.235 |

**Table S2**

Pharmacokinetic Parameters of TP in Plasma

| Group | T_1/2_  (h) | C_max_  (μg L^-1^) | | AUC_(0-t)_  (μg h L^-1^) | MRT_(0-t)_  (h) |
| --- | --- | --- | --- | --- | --- |
| TP | 0.149±0.08 | | 809.886±231.12 | 227.577±56.89 | 0.327±0.14 |
| pTP | 0.233±0.11 | | 788.896±235.25 | 360.305±151.56 | 0.580±0.65 |
| Ce6-pTP-CsA | 4.59±1.32 | | 321.844±56.56 | 332.558±66.56 | 1.789±0.69 |

**Table S3**

Quantitative Analysis of Nodule Count

| PBS | TP | pTP | Ce6-CsA | pTP-CsA | Ce6-pTP-CsA |
| --- | --- | --- | --- | --- | --- |
| 353 | 393 | 323 | 223 | 145 | 39 |
| 427 | 401 | 398 | 257 | 203 | 52 |
| 532 | 447 | 345 | 362 | 197 | 61 |
| 467 | 323 | 365 | 352 | 264 | 47 |
| 477 | 440 | 338 | 320 | 165 | 53 |

**S16**


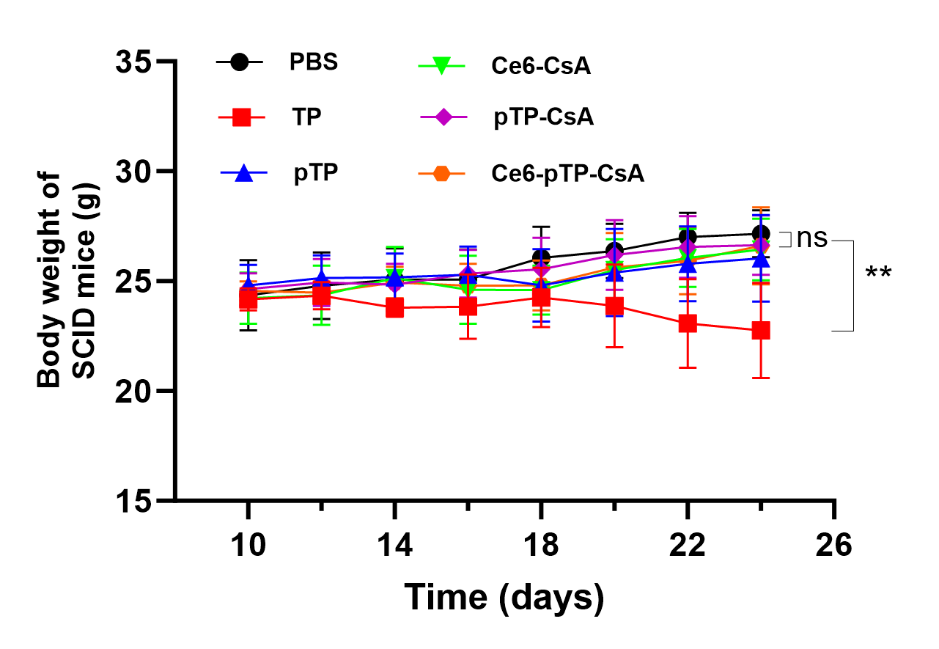


**Figure S16.** Body weight changes in each mouse group (n=5). (ns: not significant; **p < 0.01).

**S17**


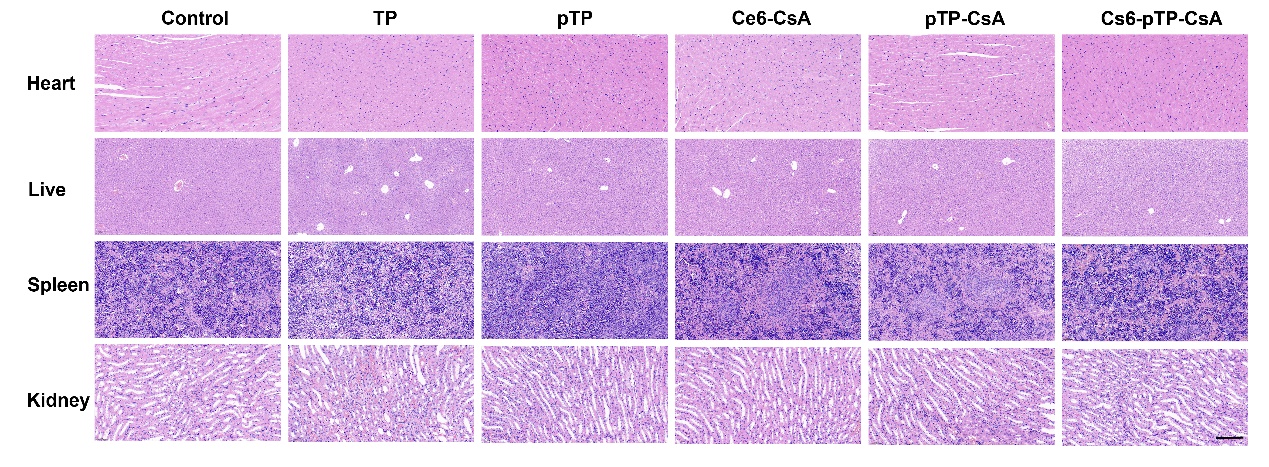


**Figure S17.** H&E staining of heart, liver, spleen, and kidney sections for each group. Scale bar: 100 μm.

**Table S4**

Hematological Analysis

| Parameters | Control | TP | pTP | | Ce6-CsA | | pTP-CsA | | Ce6-pTP-CsA | |
| --- | --- | --- | --- | --- | --- | --- | --- | --- | --- | --- |
| WBC  (10^9^·L^-1^) | 3.7±0.5 | 8.1±0.3*** | | 4.0±0.5 | | 3.3±1.0 | | 4.9±0.6 | | 4.2±0.9 |
| LympHocytes (10^9^·L^-1^) | 1.9±0.4 | 3.7±0.5** | | 2.6±0.2 | | 1.8±0.8 | | 2.9±0.4 | | 2.0±0.5 |
| Monocytes (10^9^·L^-1^) | 0.3±0.1 | 0.4±0.1 | | 0.5±0.1 | | 0.4±0.2 | | 0.3±0.2 | | 0.4±0.1 |
| Gran (10^9^·L^-1^) | 1.4±0.3 | 3.4±0.6*** | | 1.5±0.2 | | 1.6±0.3 | | 1.5±0.3 | | 1.9±0.3 |
| RBC (10^9^·L^-1^) | 7.7±0.5 | 7.7±0.2 | | 7.4±0.2 | | 8.2±0.8 | | 8.3±0.6 | | 8.1±0.4 |
| HGB (g·L^-1^) | 133.3±7.6 | 121.0±9.0 | | 130.0±5.3 | | 139.0±5.6 | | 142.3±5.6 | | 143.0±6.6 |
| PLT (10^9^·L^-1^) | 619.0±72.1 | 700.3±20.0 | | 561.3±21.5 | | 1013.0±372.0* | | 847.7±61 | | 575.7±79.1 |

**Table S5**

Biochemical Indicator Analysis

| Parameters | Control | TP | pTP | | Ce6-CsA | | pTP-CsA | Ce6-pTP-CsA |
| --- | --- | --- | --- | --- | --- | --- | --- | --- |
| ALT  (U·L^-1^) | 45.5±3.7 | 59.2±1.6*** | | 57.6±4.3** | 42.7±3.1 | 39.2±1.3 | | 42.4±3.1 |
| AST  (U·L^-1^) | 127.6±4.3 | 226.8±18.1*** | | 246.7±9.1*** | 127.0±4.3 | 122.9±7.6 | | 125.4±3.6 |
| BUN  (mg·dL^-1^) | 8.0±0.4 | 5.9±1.4* | | 8.9±0.9 | 8.1±0.8 | 8.4±0.4 | | 7.9±0.3 |
| CRFA  (μmol L^-1^) | 18.0±0.6 | 16.9±0.7 | | 21.9±5.3 | 20.4±1.4 | 19.5±1.0 | | 18.1±0.4 |
| CK-MB  (U L^-1^） | 319.8±51.7 | 355.7±59.9 | | 311.4±30.6 | 313.4±38.0 | 286.2±32.1 | | 259.4±35.7 |
